# Assessing Heavy Metal Contamination in Food: Implications for Human Health and Environmental Safety

**DOI:** 10.3390/toxics13050333

**Published:** 2025-04-23

**Authors:** Magdalena Mititelu, Sorinel Marius Neacșu, Ștefan Sebastian Busnatu, Alexandru Scafa-Udriște, Octavian Andronic, Andreea-Elena Lăcraru, Corina-Bianca Ioniță-Mîndrican, Dumitru Lupuliasa, Carolina Negrei, Gabriel Olteanu

**Affiliations:** 1 Department of Clinical Laboratory and Food Safety, Faculty of Pharmacy, “Carol Davila” University of Medicine and Pharmacy, 020956 Bucharest, Romania; magdalena.mititelu@umfcd.ro (M.M.); gabriel.olteanu@mst.umfcd.ro (G.O.); 2 Department of Pharmaceutical Technology and Biopharmacy, Faculty of Pharmacy, Carol Davila University of Medicine and Pharmacy, 020945 Bucharest, Romania; sorinel-marius.neacsu@drd.umfcd.ro (S.M.N.); dumitru.lupuliasa@umfcd.ro (D.L.); 3 Department of Cardio-Thoracic Pathology, Faculty of Medicine, “Carol Davila” University of Medicine and Pharmacy, 050474 Bucharest, Romania; stefan.busnatu@umfcd.ro; 4 General Surgery Department, “Carol Davila” University of Medicine and Pharmacy, 050474 Bucharest, Romania; 5 Innovation and eHealth Center, “Carol Davila” University of Medicine and Pharmacy, 050474 Bucharest, Romania; 6 Department of Cardiology, Emergency Hospital “Bagdasar-Arseni”, 050474 Bucharest, Romania; andreea.lacraru@umfcd.ro; 7 Department of Toxicology, Faculty of Pharmacy, “Carol Davila” University of Medicine and Pharmacy, 020945 Bucharest, Romania; corina-bianca.ionita-mindrican@drd.umfcd.ro (C.-B.I.-M.); carolina.negrei@umfcd.ro (C.N.)

**Keywords:** food contamination, food toxicity, mercury, lead, cadmium, arsenic, tin, food security

## Abstract

Ensuring food security is essential for achieving sustainable global development, requiring a balance between sufficient food production and maintaining its safety and nutritional value. However, this objective faces considerable challenges due to the infiltration of toxic metal species into the food supply. Heavy metals and metalloids, depending on their molecular form and daily dose, exhibit varying degrees of toxicity, making the precise identification of their species essential for assessing their impact on human health and the environment. This study focuses on identifying the primary anthropogenic sources and dissemination pathways of heavy metal pollutants, with an emphasis on their speciation and bioavailability. It examines how toxic metal species, such as Pb^2+^, Cd^2+^, Hg^2+^, and various arsenic species (AsIII and AsV), infiltrate ecosystems, bioaccumulate within the food chain, and ultimately compromise food safety and nutritional value. Furthermore, the research explores the physiological and biochemical disruptions caused by these toxic metal species, including the displacement of essential ions from enzymatic active sites and transport proteins due to competitive binding by pollutants, oxidative stress induced by reactive oxygen species generation, and cellular dysfunction affecting metabolic pathways and signaling cascades, all of which contribute to both chronic and acute health conditions. By providing a detailed analysis of exposure routes and toxicological processes, this paper highlights the far-reaching consequences of heavy metal contamination on public health and agricultural sustainability. Special attention is given to the need for precise terminology, as the toxicity of metals is inherently linked to their daily dose and chemical species rather than their elemental form. Finally, this study advocates for integrated, multidisciplinary strategies aimed at mitigating these risks, enhancing ecosystem stability, and ensuring long-term food security in the face of environmental challenges.

## 1. Introduction

Food security has become a critical priority for sustainable global development, encompassing both the quantity and quality of food available. In recent decades, the exceedance of toxic contaminants beyond threshold values in crops has posed significant threats to food safety and human health. Among these, specific heavy metal and metalloid species are particularly concerning due to their potential to alter human metabolic processes through chronic low-level exposure (e.g., 0.5–5 µg/kg/day), contributing to increased morbidity observed in both human biomonitoring studies and animal models [1]. Chemically, the term heavy metals broadly refer to approximately 40 elements with a density greater than 5 g/cm^3^. However, their toxicity depends of chemical species and daily dose. Highly toxic metal species such as methylmercury (MeHg), cadmium ions (Cd^2+^), lead ions (Pb^2+^), and inorganic arsenic species (AsIII and AsV) can bind to enzymes in mammalian cells (e.g., hepatic or neuronal), such as glutathione reductase or δ-aminolevulinic acid dehydratase, or interact with key cellular components, leading to enzymatic inhibition after exposure periods ranging from hours (in vitro) to weeks (in vivo models), disruption of redox homeostasis, interference with ion transport mechanisms, and impairment of DNA repair processes, ultimately contributing to cytotoxicity and systemic toxicity [2,3]. Their ability to bioaccumulate within the food chain amplifies their risk to human health and environmental stability.

Major sources of heavy metal contamination include industrial emissions (such as those from mining, smelting, and manufacturing), agricultural practices (including the use of phosphate fertilizers containing cadmium and pesticides with arsenic-based compounds), and urban waste (such as electronic waste and untreated sewage), which introduce these toxic metal species into soil, water, and air. Once present in these ecosystems, they infiltrate food systems, ultimately compromising the safety of products intended for human and animal consumption. Addressing the risks associated with heavy metal contamination requires the precise identification of toxic metal species and their pathways within the environment to implement effective mitigation strategies [4,5].

**Figure 1:**
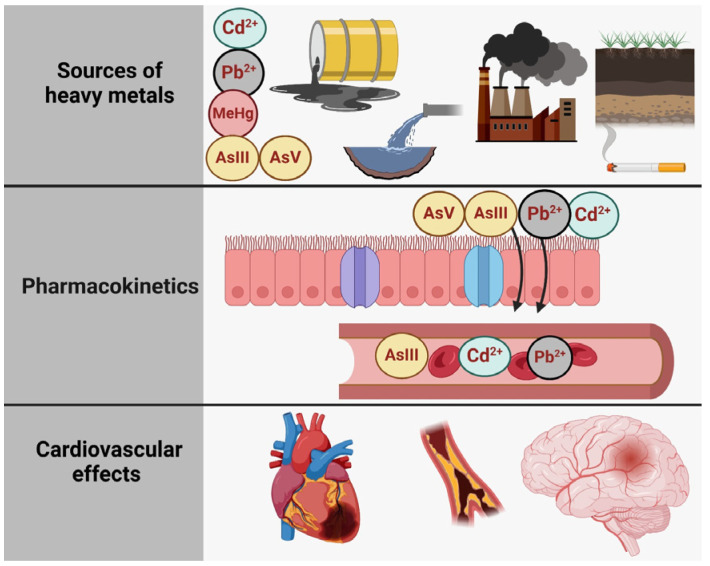
The primary sources of heavy metal contamination, the pharmacokinetics of heavy metals, and their effects on the cardiovascular and cerebrovascular systems. Created with BioRender.com (accessed on 25 October 2024).

Figure 1 illustrates the relationship between toxic metal species exposure and health outcomes, structured into three main sections: sources of exposure, pharmacokinetics, and adverse effects of the heart, the brain, and the bones [6]. The first section (A. Sources of Exposure) presents the various pathways through which individuals can be exposed to specific toxic metal species, including Pb^2+^ (lead), Cd^2+^ (cadmium), and inorganic arsenic (AsIII and AsV). These contaminants originate from industrial pollution (depicted as factories and emissions), contaminated water, vegetables grown in polluted soil, tobacco smoke, and even certain consumer products, such as candles with metallic wicks [7]. The second section (B. Pharmacokinetics) outlines the processes of absorption, distribution, metabolism, and excretion of these metal species. Once absorbed through the respiratory and digestive tracts, they are transported systemically via the bloodstream. Pb^2+^ and Cd^2+^ tend to accumulate in tissues, particularly in bones and kidneys, while inorganic arsenic species (AIII and AsV) undergo biotransformation via methylation into monomethylarsonic acid (MMA) and dimethylarsinic acid (DMA), facilitating their excretion in urine. Each toxic metal species follows a distinct metabolic pathway, influencing its persistence and toxicity in the body. Finally, the third section (C. Cardiovascular Outcomes) highlights the link between exposure to these toxic metal species and cardiovascular or other diseases. Chronic exposure to Pb^2+^, Cd^2+^, and inorganic arsenic is associated with conditions such as ischemic heart disease, stroke, and peripheral artery disease, underscoring the significant public health risks posed by environmental contamination.

The increasing challenges of food security have become a significant global concern, largely due to their direct implications for human health. Certain toxic metal species, including inorganic arsenic (AIII and AsV), lead ions (Pb^2+^), cadmium ions (Cd^2+^), and mercury species such as methylmercury (MeHg) and mercuric ions (Hg^2+^), are non-essential for biological and metabolic functions [1]. Due to their significant health risks, these toxic metal species are classified as priority hazardous substances by regulatory agencies such as the United States Environmental Protection Agency (EPA) and the Agency for Toxic Substances and Disease Registry (ATSDR) [8]. Additionally, various international regulatory bodies, including the European Food Safety Authority (EFSA) and the U.S. Food and Drug Administration (FDA), have established strict guidelines and maximum allowable limits for toxic metal residues in food to minimize human exposure. These regulations aim to protect public health by monitoring contamination levels, enforcing safety standards, and implementing risk assessment protocols to mitigate the adverse effects of toxic metal species in the food supply (Table 1).

Heavy metal contamination of drinking water represents a significant global public health issue, as water serves as a primary vector for human exposure to toxic elements such as lead (Pb^2+^), arsenic (AsIII and AsV), cadmium (Cd^2+^), and mercury (Hg^2+^). These contaminants originate from both natural sources, such as the geochemical composition of aquifers, and anthropogenic activities, including industrial discharge, mining operations, agricultural runoff, and the degradation of water distribution infrastructure. The persistence and bioaccumulative nature of heavy metals make their presence in drinking water particularly concerning, as even low-level, chronic exposure has been associated with severe health consequences, including neurotoxicity, nephrotoxicity, hepatotoxicity, immunosuppression, and carcinogenic effects. Lead exposure, for instance, has been extensively linked to cognitive impairments, developmental delays in children, and cardiovascular diseases in adults. Arsenic contamination, a major concern in regions such as Bangladesh and parts of South Asia, has been implicated in arsenicosis, a condition characterized by skin lesions, peripheral neuropathy, and increased risk of multiple cancers. Similarly, cadmium accumulation due to prolonged ingestion of contaminated water contributes to renal dysfunction, osteoporosis, and metabolic disturbances affecting calcium homeostasis.

Many studies analyzing drinking water samples from various regions have highlighted the severity of heavy metal contamination. Arsenic contamination of groundwater in Bangladesh poses a significant public health challenge, with numerous studies highlighting its severity. A comprehensive survey conducted by the British Geological Survey (BGS) and the Department of Public Health Engineering (DPHE) between 1998 and 2001 analyzed 2022 well water samples across 41 of Bangladesh’s 64 districts. The findings revealed that 51% of the samples exceeded the World Health Organization’s (WHO) guideline value of 10 µg/L for arsenic in drinking water, and 35% surpassed Bangladesh’s national standard of 50 µg/L. Alarmingly, 25% of the samples contained arsenic concentrations above 100 µg/L, with some wells registering levels as high as 1000 µg/L [17].

Further investigations have pinpointed regions such as the southwestern and northeastern areas of Bangladesh as particularly affected by high arsenic concentrations. For instance, the BGS and DPHE study found that only 4.6% of 326 deep groundwater samples collected from these regions had arsenic concentrations greater than 10 µg/L. However, this sample set was limited and may not fully represent the broader contamination issue [18,19].

These elevated arsenic levels have profound health implications, including skin lesions, internal cancers, and developmental effects. Addressing this crisis requires a multifaceted approach, including the provision of safe drinking water alternatives, public awareness campaigns, and the implementation of arsenic removal technologies.

In Punjab, India, the discharge of industrial effluents from textile and electroplating industries has led to significant contamination of water sources with heavy metals, notably, cadmium. Studies have reported cadmium concentrations in water samples from this region ranging from 0.01 to 0.15 mg/L, which substantially exceeds the World Health Organization’s (WHO) permissible limit of 0.003 mg/L. This elevated cadmium presence is primarily attributed to the effluents released by these industries, highlighting a critical environmental and public health concern [20].

In Flint, Michigan, a significant public health crisis unfolded between 2014 and 2015 when the city’s water source was switched to the Flint River without implementing necessary corrosion control treatments. This oversight led to the leaching of lead from aging lead-based pipelines into the drinking water supply. In one notable instance, water sampled from a Flint household exhibited a lead concentration of 13,000 parts per billion (ppb), a level far exceeding the U.S. Environmental Protection Agency’s (EPA) action threshold of 15 ppb [21,22]. This catastrophic event underscores the critical importance of proper water treatment protocols and infrastructure maintenance to prevent such hazardous contamination.

The assertion that a nationwide survey in China found mercury contamination exceeding the WHO guideline of 1 µg/L in over 10% of sampled water sources, with concentrations reaching up to 5 µg/L, is supported by several studies. For instance, a comprehensive review highlighted that the total mercury (THg) concentration of 1 µg/L in water is considered the upper limit for drinking water by organizations such as the World Health Organization (WHO), Australia, and Canada [23]. This standard is widely recognized and applied in assessing water quality.

Regulatory agencies, including the WHO, EPA, and EFSA, have established maximum allowable limits for heavy metals in drinking water (Table 1). However, compliance remains a significant challenge, particularly in regions with inadequate monitoring systems and insufficient resources for large-scale water purification initiatives. Conventional treatment methods, such as activated carbon filtration, ion-exchange resins, and reverse osmosis, have demonstrated efficacy in heavy metal removal but are often limited by high operational costs and maintenance requirements. Emerging remediation technologies, including nanotechnology-based adsorbents, electrocoagulation, and biological filtration using microbial and algal bioadsorbents, offer promising alternatives but require further optimization for large-scale deployment. Moreover, household-level interventions, such as the use of certified water filters, regular well water testing, and adherence to safety guidelines regarding groundwater consumption, are critical in mitigating health risks associated with contaminated drinking water.

Addressing heavy metal contamination in drinking water necessitates a comprehensive and interdisciplinary approach, integrating environmental monitoring, technological advancements, public health interventions, and policy-driven initiatives. Strategies such as the replacement of lead-containing plumbing materials, stricter regulations on industrial effluents, and improved agricultural practices to minimize heavy metal runoff are essential components of a long-term solution. Additionally, the implementation of sustainable water sourcing methods, such as rainwater harvesting and deep aquifer extraction in high-risk areas, may reduce dependency on contaminated groundwater supplies. By incorporating detailed discussions on regional data analyses of drinking water contamination into the broader discourse on heavy metal exposure, research efforts can provide a more holistic understanding of the issue while emphasizing practical mitigation strategies aimed at safeguarding public health.

Regulatory thresholds for heavy metals in food and drinking water are pivotal in mitigating the risks associated with chronic exposure to toxic metals, particularly in the context of food safety and public health. International regulatory bodies, including the European Union and the United States, have implemented stringent guidelines to limit the concentrations of hazardous metals in various food products. In the European Union, for instance, the maximum allowable concentration of cadmium in wheat is set at 100 ppb, which is designed to prevent market contamination and protect consumers from potential health risks, including renal and bone damage associated with long-term cadmium exposure (European Commission, 2006) [24]. Similarly, the FDA enforces limits for metals like Pb, Hg, and As in food, with specific regulations in place for foods intended for vulnerable populations. For example, the FDA limits lead in candy products to 0.1 ppm, and arsenic in apple juice to 10 ppb, acknowledging the heightened sensitivity of children to these toxicants (FDA, 2013) [25]. These regulatory standards play an important role in the global food trade, as exceeding these limits typically leads to market rejection, further incentivizing compliance by food producers.

One of the most alarming emerging concerns is the presence of toxic metals in baby foods, particularly those containing high levels of lead, cadmium, and arsenic. According to a study published in Spectroscopy (2021) [26], there has been a growing awareness of this issue, especially after the U.S. Congress directed the FDA to investigate the presence of these metals in baby foods. The study revealed that certain baby food products, such as rice-based cereals, may exceed the safety thresholds for arsenic, with some containing levels up to 150 ppb, far above the FDA’s recommended limit of 10 ppb for drinking water (FDA, 2013) [27]. The risk posed by heavy metal contamination in baby foods is particularly concerning due to the heightened vulnerability of infants, whose developing systems are more susceptible to the neurotoxic effects of Pb^2+^ and Hg, and the carcinogenic effects of inorganic arsenic compounds (AsIII and AsV). The growing body of research and regulatory focus on this issue underscores the importance of establishing more robust regulatory frameworks and improving monitoring systems to safeguard the health of the most vulnerable populations. Recent calls for stricter regulatory measures, such as lowering the permissible limits for metals in foods consumed by infants and children, are gaining momentum, highlighting the need for more comprehensive food safety standards globally.

Soil, as the fundamental medium for food crop growth, is particularly vulnerable to contamination by heavy metal species. These pollutants originate from both point sources—such as energy-intensive industries including coal-fired power plants, gold mining, chlor-alkali chemical production, metal smelting, galvanization, textile and leather processing, and electronic waste disposal—and non-point sources, including soil and sediment erosion, agricultural runoff, and unregulated storage practices [28]. Beyond their direct toxicity to humans, heavy metal ions such as Cd^2+^ and Pb^2+^ disrupt soil ecosystems by altering microbial community structure and enzymatic activity, thereby impairing nutrient cycling and soil fertility. While certain microorganisms can tolerate elevated metal concentrations through mechanisms like efflux pumps and bioaccumulation, others are highly sensitive, leading to shifts in microbial diversity and function. Moreover, Cd^2+^ and Pb^2+^ readily accumulate in agricultural soils, increasing their bioavailability and facilitating uptake by food crops, which poses serious risks to food safety and human health. These effects highlight the necessity for rigorous monitoring and remediation strategies to maintain soil productivity and ensure crop quality [29].

In vivo and in vitro experimental studies have repeatedly demonstrated that exposure to toxic metal species disrupts key metabolic pathways, including the oxidative stress response, mitochondrial respiration, and essential enzyme functions involved in detoxification (e.g., glutathione metabolism) and energy production (e.g., glycolysis and the Krebs cycle). These disruptions can impair cellular homeostasis, promote inflammation, and contribute to the pathogenesis of various diseases [30]. Mechanisms such as endothelial dysfunction, chronic inflammation, hypertension, nephrotoxicity, oxidative stress, disruptions in cardiac electrophysiology, cardiotoxicity, and epigenetic modifications are frequently observed in individuals after chronic exposure—or, less commonly, acute exposure due to industrial accidents—to toxic metal species [6]. Specific toxic metal species, including Pb^2+^, Hg^2+^ and MeHg, Cd^2+^, and inorganic arsenic (AsIII and AsV), can bioaccumulate in tissues, disrupting cellular homeostasis through multiple pathways. These effects include the inhibition of enzymatic activity, such as the Cd^2+^-mediated inhibition of superoxide dismutase (SOD), leading to impaired antioxidant defense mechanisms. Additionally, toxic metal species induce oxidative stress by generating reactive oxygen species (ROS), which can cause both direct DNA damage, such as strand breaks and base modifications, and indirect DNA damage through lipid peroxidation and protein oxidation. Collectively, these disruptions contribute to neurotoxicity, kidney dysfunction, cardiovascular disorders, and immune system impairment. Chronic exposure has been linked to developmental delays in children, carcinogenic effects, and endocrine disruptions [31]. Additionally, toxic metal species such as Cd^2+^ and Pb^2+^ can disrupt essential nutrient metabolism by interfering with the absorption, transport, and utilization of key minerals, including Ca^2+^, Zn^2+^, and Fe^2+^ [32,33]. For example, Cd^2+^ competes with Zn^2+^ for binding sites on metallothioneins, leading to zinc deficiency and impaired enzymatic function, while Pb^2+^ disrupts calcium homeostasis by mimicking Ca^2+^, affecting neuronal signaling and bone mineralization. These disruptions contribute to oxidative stress, systemic inflammation, and the development of metabolic disorders. The severity of these effects depends on several factors, including the dose and duration of exposure, the chemical form of the metal, and individual susceptibility factors such as age, genetics, and overall health status. Addressing heavy metal toxicity requires a comprehensive understanding of metal speciation, bioavailability, and long-term health consequences.

The primary and most prevalent mechanism through which toxic metal species exert their detrimental effects involves the disruption of metalloproteins and enzyme function by competing with or displacing essential divalent ions such as Zn^2+^ from SOD, Ca^2+^ from calmodulin, and Cu^2+^ from cytochrome c oxidase (CcO). Thus, Cd^2+^ can replace Zn^2+^ in zinc-dependent enzymes and transcription factors, impairing their catalytic activity and regulatory functions [33]. Similarly, Pb^2+^ can interfere with Ca^2+^ signaling by mimicking its role in neuronal and cellular processes, by displacing Ca^2+^ in synaptic vesicle fusion. These disruptions compromise key biological functions, such as redox regulation, neurotransmission, and gene expression, ultimately leading to cellular oxidative stress, impaired signal transduction, and apoptosis; these are processes that underlie the pathogenesis of various chronic diseases, including neurodegeneration, cancer, and developmental disorders. This ion substitution disrupts critical metabolic pathways such as oxidative phosphorylation, calcium signaling, and antioxidant defense systems. The replacement of essential ions like Zn^2+^ or Ca^2+^ by toxic metals such as Cd^2+^ or Pb^2+^ can impair mitochondrial energy production, inhibit enzymatic activity (e.g., of SOD or ATPases), and dysregulate intracellular signaling. As a result, structural alterations may occur in organ systems such as the kidney, liver, and brain, where cumulative damage results in fibrosis, neurodegeneration, or tissue atrophy. Physiological responses to stimuli, such as hormonal signaling, immune activation, or neuronal excitability, may become inappropriate or exaggerated, contributing to conditions such as hypertension, immunosuppression, or seizures [32]. In severe cases, these cascading effects can culminate in life-threatening outcomes (e.g., renal failure, cardiac arrhythmias, systemic inflammatory responses) or contribute to a cascade of toxic effects, further exacerbating metabolic imbalances and increasing disease susceptibility [6].

This review examines the risks associated with heavy metal contamination in the food chain, emphasizing the role of metal speciation and bioavailability in determining toxicity. In addition to assessing contamination sources and exposure pathways, the study explores strategies for mitigating health risks through dietary interventions and preventive measures. A key focus is placed on protective dietary factors, including specific food choices that can reduce heavy metal absorption and enhance detoxification processes. Furthermore, the review discusses practical approaches to minimizing exposure, such as contamination reduction strategies, food monitoring programs, and consumer guidance on selecting less contaminated food products. By integrating toxicological insights with nutritional and public health perspectives, this study provides a comprehensive framework for reducing the impact of heavy metals on human health.

## 2. Materials and Methods

This review study was conducted through a comprehensive analysis of the scientific literature on food contamination with heavy metals and their impact on human health. The research included articles retrieved from reputable scientific databases such as PubMed, Scopus, Web of Science, and Google Scholar. Relevant keywords such as “heavy metals”, “food contamination”, “food toxicity”, “mercury”, “lead”, “cadmium”, “arsenic”, and “tin” were used in combination with logical operators to refine search results.

Only articles addressing contamination sources, pathophysiological mechanisms, and health effects were included. The analysis of selected articles focused on identifying sources of dietary exposure, evaluating the mechanisms through which heavy metals exert toxic effects, and determining specific, actionable measures to mitigate risks to individual and public health.

The collected information was synthesized to provide a comprehensive overview of the role of heavy metals in health deterioration. Emphasis was placed on experimental, observational, and, also, review studies exploring cardiovascular, neurological, and metabolic effects, highlighting the molecular and cellular mechanisms involved.

The results of this analysis offer an integrated perspective on the issue of food contamination with heavy metals, underscoring the urgent need for effective preventive and remedial measures to safeguard public health.

### 2.1. Contamination Sources

The primary sources of toxic metal species in soil and agriculture stem from both natural and anthropogenic activities. Natural sources include the weathering of metal-rich rocks, volcanic emissions, and geothermal activity, which contribute to baseline levels of toxic metals in the environment. However, human activities have significantly amplified contamination levels, posing a substantial risk to agricultural productivity and food safety. Anthropogenic sources of toxic metal contamination in soil include atmospheric deposition from industrial emissions, irrigation with wastewater or contaminated water, and the use of fertilizers, pesticides, and herbicides that contain metal impurities [34]. Phosphate-based fertilizers are particularly concerning, as they often contain cadmium (Cd^2+^) and lead (Pb^2+^) as byproducts, which gradually accumulate in agricultural soils. Sewage sludge amendments and the disposal of metal-rich industrial waste further exacerbate contamination levels, introducing harmful species such as arsenic (AsIII and AsV), mercury (Hg^2+^ and MeHg), and cadmium [35]. Other significant contributors include coal ash residues, emissions from petrochemical industries, and historical use of leaded gasoline and paints, which have led to long-term soil contamination in urban and peri-urban agricultural areas. The repeated application of fertilizers and organic amendments can also contribute to bioavailable forms of toxic metals, increasing their uptake by plants over time. These contaminants infiltrate crops primarily through root absorption from polluted soils, where metal species bind to clays and organic matter, influencing their bioavailability. Additionally, atmospheric deposition can lead to direct contamination of plant surfaces, particularly in regions affected by industrial emissions or urban pollution [36]. Once inside plant tissues, these toxic metal species can bioaccumulate, posing direct risks to human health through dietary exposure [37].

The persistence of these contaminants in soil and their capacity for bioaccumulation in food crops underscore the urgent need for stringent monitoring, sustainable agricultural practices, and effective soil remediation strategies to minimize human exposure and ensure food safety [38].

Industrial and vehicular emissions, particularly from diesel combustion, are major sources of heavy metal contamination, releasing airborne particulates containing lead ions (Pb^2+^), cadmium ions (Cd^2+^), arsenic ions (AsIII and AsV), and mercury ions (Hg^2+^), which eventually settle into the soil and infiltrate the food chain. Among industrial sources, coal-fired power plants are particularly significant, releasing substantial amounts of mercury (Hg) in various forms, including elemental mercury (Hg^0^) and oxidized mercury (Hg^2+^). For example, global coal combustion is estimated to emit approximately 2000 tonnes of mercury annually, with regional emissions varying based on coal type and pollution control measures. These mercury deposits can accumulate in agricultural soils, posing risks to food safety and ecosystem health [39]. Studies have shown that prolonged consumption of crops such as leafy vegetables (e.g., lettuce, amaranth, water spinach) and cereals (e.g., rice) grown in Hg-contaminated soils poses severe health risks, including neurotoxicity, kidney dysfunction, and cardiovascular disorders. Ensuring the quality and safety of food crops is especially challenging because agricultural irrigation water is often inadequately treated [40]. Contaminated water sources, whether from industrial discharge, urban runoff, or mining activities, introduce heavy metal species such as Pb^2+^, Cd^2+^, AsIII, and Hg^2+^ into farmland. This leads to metal uptake by crops and subsequent bioaccumulation in edible plant parts, increasing dietary exposure risks. Irrigation with untreated or poorly treated water fails to safeguard agricultural land from contamination, making effective water treatment and monitoring systems crucial for food safety [41].

Identifying soil contaminants and their sources is a critical research priority due to their direct impact on human health, ecosystem stability, and agricultural productivity. In addition to conventional heavy metal pollution, emerging concerns such as the presence of engineered nanoparticles (NPs) in soil have gained attention. Copper oxide nanoparticles (CuO NPs), typically ranging in diameter from 10 to 50 nm, have been studied for their nanotoxicity and potential application as nanofertilizers. According to Ibrahim et al. (2022), exposure to CuO NPs at concentrations of 50–200 mg/L for 7–14 days led to both toxic and stimulatory effects on *Triticum aestivum* (wheat) plants, depending on the dose. At lower concentrations (≤50 mg/L), CuO NPs enhanced plant growth and chlorophyll content, whereas higher concentrations (≥100 mg/L) induced oxidative stress, reduced photosynthetic efficiency, and impaired root development due to excessive reactive oxygen species (ROS) generation. The small size and high reactivity of CuO NPs facilitate their uptake and translocation within plant tissues, influencing nutrient homeostasis and overall plant health [42]. The ingestion of crops exposed to nanomaterials may also pose unforeseen risks to human health, warranting further toxicological investigations [1].

Remediating heavy metal-contaminated soils is essential for reducing associated health and environmental risks, restoring land for sustainable agricultural use, and strengthening global food security. Strategies such as phytoremediation (using hyperaccumulator plants), soil amendments (e.g., biochar, zeolites, organic matter), and advanced water filtration technologies can mitigate contamination levels, ensuring safer agricultural production and protecting long-term food sustainability [43].

**Figure 2:**
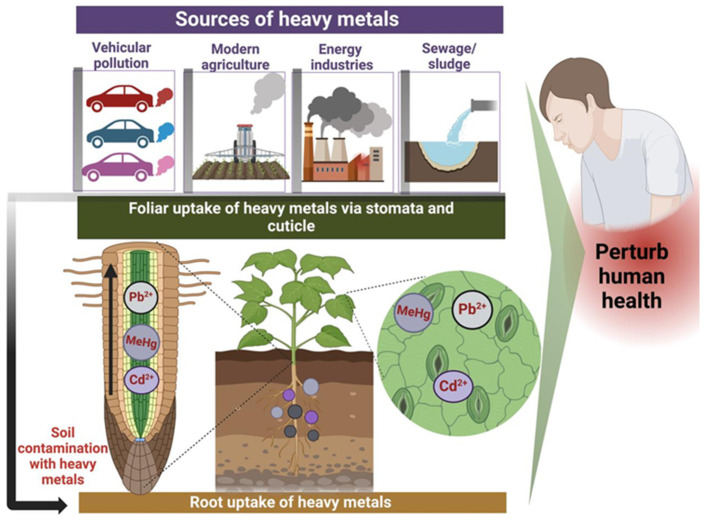
Natural and anthropogenic sources of heavy metal contamination in food crops, along with their entry mechanisms (via stomata/cuticles), and the resulting impact on biota and human health. Created with BioRender.com (accessed on 25 October 2024).

Soils serve as a primary reservoir for heavy metals that are introduced into the environment through anthropogenic activities, such as industrial emissions, agricultural practices, and waste disposal (Figure 2). Unlike organic pollutants, which can be biodegraded into carbon dioxide and water through microbial processes, heavy metals cannot be broken down but instead persist in the environment, undergoing transformation through processes such as oxidation, reduction, complexation, and adsorption. As a result, these metals persist in the environment for extended periods, accumulating in soils, water, and organisms [44]. This persistence, coupled with their toxicological effects, makes heavy metals a significant environmental and public health concern. As a result, once deposited, their total concentrations in soil persist over time, continuously posing a long-term threat to environmental and human health [1].

Despite their persistence, heavy metals can undergo various physicochemical transformations, including oxidation-reduction reactions (e.g., Fe^2+^/Fe^3+^, AsIII/AsV, or Cr^3+^/Cr^6+^), complexation with organic ligands (such as humic acids, amino acids, or thiol groups) or inorganic anions (like Cl^−^, SO_4_^2−^, or PO_4_^3−^), and adsorption onto solid surfaces, such as clay minerals, iron (Fe^3+^) and manganese (Mn^4+^) oxides, or organic matter. These processes, driven by electrostatic interactions, ion exchange, or surface precipitation, influence the speciation, mobility, and bioavailability of heavy metals in environmental systems over time. These processes can alter the toxicity and mobility of heavy metals in soil, influencing their potential to enter the food chain. Toxic metal contamination in soil can have severe consequences for both ecological systems and human health through multiple exposure pathways. Direct contact with contaminated soil, including skin exposure, can result in harmful absorption of toxic metals, especially in high-risk areas like urban gardens or recreational spaces. Soil-to-plant transfer represents a major route for these metals to enter the food chain, where they bioaccumulate in edible plant parts, ultimately affecting human and animal health [45]. Furthermore, animal consumption of contaminated plants or grazing in polluted areas can lead to further bioaccumulation in the food chain. Contaminated groundwater, used for irrigation or drinking, can also pose a significant health risk, as metals leach into water sources and increase human exposure [46].

Beyond direct health risks, the presence of heavy metals in soil can significantly reduce crop quality, compromising both the safety and marketability of agricultural products. Phytotoxic effects, including stunted growth, reduced yields, and altered nutrient uptake, can diminish crop productivity, thereby affecting food supply and economic viability for farmers [47]. These effects can also limit land availability for safe agricultural use, leading to broader socio-economic challenges such as food insecurity and disputes over land ownership and access to clean water resources [48].

Concerns over food quality are escalating globally, primarily due to the detection of toxic elements in a wide range of food products. This has prompted extensive research into the toxicological impacts these contaminants have on human health, with a particular focus on heavy metals, which are among the most significant environmental pollutants. Heavy metals are of particular concern in aquatic ecosystems due to their high toxicity and potential for bioaccumulation in marine organisms. This bioaccumulation process can lead to dangerous concentrations of metals in seafood, posing substantial risks to both environmental health and food safety [49].

Heavy metal contamination in aquatic environments originates from both natural and anthropogenic sources. Naturally, these metals can enter water bodies through processes such as the weathering of rocks, volcanic eruptions, and soil erosion, which release trace metal ions of Hg and Cd or metalloid as As into rivers, lakes, and oceans. While these natural sources contribute to background metal levels, human activities are the primary contributors to elevated concentrations in aquatic systems [37]. Industrial discharges from activities such as mining, smelting, and manufacturing processes release significant quantities of toxic metals into water systems. For example, mercury ions (Hg^2+^ and Hg^0^) are often discharged by gold mining operations, while lead (Pb^2+^) and cadmium (Cd^2+^) ions can be introduced through smelting and metal production. Wastewater from factories further contributes to pollution, as many industries use or produce heavy metal species as by-products. Agricultural practices also play a key role in introducing heavy metal species into water bodies. The use of chemical fertilizers and pesticides can result in metals like ions of cadmium and arsenic species such as As(III) and As(V) being leached into nearby water sources through runoff or irrigation. Additionally, the use of contaminated irrigation water is a growing concern in regions where industrial and agricultural runoff enters water supplies, exacerbating contamination. Urban runoff is another significant contributor to heavy metal pollution, with vehicle emissions, construction debris, and improper waste disposal adding to the metal load in waterways [50]. Accidental oil spills and the dumping of electronic and medical waste also release hazardous metals, such as lead from batteries and mercury from electrical components, into aquatic environments [51].

These diverse sources of contamination not only threaten water quality but also have profound effects on aquatic organisms, which may accumulate harmful levels of metals in their tissues. This bioaccumulation poses indirect risks to human health through the consumption of contaminated seafood, which can lead to a variety of health issues, such as neurological disorders, kidney damage, and cancer. Furthermore, heavy metal contamination can disrupt aquatic ecosystems by affecting the reproductive health and survival of marine species, ultimately altering food webs and biodiversity [52].

As these toxic metal ions accumulate in marine life, their concentrations increase as they increase along the food chain, a process known as biomagnification. This results in human consumption of contaminated seafood, leading to exposure to significantly higher concentrations of mercury species (such as methylmercury, MeHg^+^), lead (Pb^2+^), and cadmium (Cd^2+^), which contributes to a range of health problems. The specific metal ions of concern include methylmercury (MeHg) exposure, which is most commonly linked to neurological disorders. It can cause memory loss, cognitive impairment, tremors, and nervous system damage, especially in high levels of methylmercury, which bioaccumulates in fish and shellfish [53]. Long-term exposure can result in permanent neurological damage, particularly in developing fetuses and young children. Cd^2+^ is associated with kidney and liver damage, particularly in individuals who consume contaminated seafood over long periods. It also causes bone demineralization and respiratory issues if inhaled. Chronic exposure to cadmium can result in kidney dysfunction and the development of renal diseases [54]. Pb^2+^ exposure is known to cause cardiovascular diseases, hypertension, and developmental issues, particularly in children. Lead can disrupt various biological systems, including the nervous, cardiovascular, and renal systems. It is also linked to learning disabilities, reduced IQ, and behavioral problems in children [55]. Arsenic, particularly in its inorganic forms (AsIII and AsV), is a well-established carcinogen. Chronic exposure increases the risk of cancers in the bladder, lungs, and skin, as well as developmental effects and metabolic disruptions. It can also impair the function of the cardiovascular and respiratory systems [56].

Toxic metal species, including lead (Pb^2+^), cadmium (Cd^2+^), mercury (Hg^2+^), and arsenic (AsIII), exert their detrimental effects on mammalian metabolism through a diverse array of biomolecular mechanisms, which contribute to a wide spectrum of pathological conditions. One of the most prevalent and fundamental mechanisms of toxicity is the interaction of these metals with critical cellular macromolecules, such as proteins, lipids, and nucleic acids, which disrupts normal cellular homeostasis. These metals can bind to thiol groups (-SH) on proteins, altering their conformation and, in many cases, causing enzyme inhibition, aberrant signaling, and interference with normal cellular functions [57]. Cadmium, for example, mimics essential divalent metals such as Zn^2+^ and Ca^2+^ by displacing Zn^2+^ from metallothioneins and Ca^2+^ from calmodulin in neuronal cells, which results in disrupted Ca^2+^ signaling and the activation of pathways related to oxidative stress. This oxidative stress is primarily mediated by the generation of reactive oxygen species (ROS) that damage cellular structures, induce lipid peroxidation, and disrupt mitochondrial function, leading to apoptosis and necrosis. This has been demonstrated in in vitro studies using human cell lines (e.g., HepG2, SH-SY5Y) at concentrations of 1–10 µM, and in rodent models with chronic exposure over 8–12 weeks. Mercury, a potent neurotoxicant, forms strong covalent bonds with thiol groups on proteins, causing structural changes that impair neurotransmission and contribute to neurodegeneration. This disruption extends to mitochondrial dysfunction, leading to a reduction in ATP production, which further exacerbates cellular stress and compromises cellular energy homeostasis. Arsenic, a known carcinogen, interferes with key DNA repair mechanisms by disrupting the function of repair proteins such as RAD51, which results in the accumulation of DNA double-strand breaks and genomic instability. This effect is primarily associated with inorganic arsenic species (AsIII and AsV). Moreover, inorganic arsenic species induce epigenetic modifications, including DNA methylation changes, that alter gene expression patterns and promote the progression of cancer. Lead ions (Pb^2+^), in particular, disrupt heme biosynthesis by inhibiting porphobilinogen deaminase and ferrochelatase, enzymes involved in the production of heme, resulting in anemia and impairing oxygen transport capacity. In addition, lead exposure leads to the activation of inflammatory cascades through the NF-κB pathway, promoting chronic inflammation, which is implicated in the pathogenesis of cardiovascular and renal diseases [57,58]. These metals can also directly modulate transcriptional activity, altering the expression of genes involved in cell cycle regulation, apoptosis, and immune response, thereby contributing to carcinogenesis and the progression of degenerative diseases. Collectively, the molecular perturbations induced by heavy metals lead to a cascade of downstream effects, including oxidative stress, mitochondrial dysfunction, inflammation, and altered cell signaling, which underlie the systemic toxicity observed in target organs such as the liver, kidneys, cardiovascular system, and nervous system [58,59]. These cellular and molecular disruptions not only compromise individual organ function but also synergize to exacerbate multi-organ damage, presenting a significant public health concern in the context of environmental exposure to toxic metals. The complexity and diversity of these molecular mechanisms highlight the necessity for stringent regulatory frameworks, continuous biomonitoring, and advanced mitigation strategies to limit human exposure to these pervasive environmental contaminants and reduce their detrimental impact on human health and ecological integrity.

Arsenicosis, a disease resulting from prolonged exposure to arsenic-contaminated drinking water, remains a significant public health issue in regions like Bangladesh, India, and parts of Southeast Asia, where groundwater arsenic levels frequently exceed the World Health Organization (WHO) guideline of 10 µg/L. In Bangladesh, studies have shown that nearly 35 million people are at risk of chronic exposure to As(III) and As(V) due to contaminated drinking water sources, with arsenic concentrations in some wells exceeding 300 µg/L. Long-term exposure to arsenic has been linked to several adverse health outcomes, including skin lesions, respiratory issues, cardiovascular diseases, and cancers of the skin, lungs, and bladder [60]. In addition to this, the teratogenic and neurotoxic effects of arsenic on children’s development are also well-documented, further emphasizing the widespread and ongoing public health challenge posed by arsenic contamination [61].

Plumbism, or lead poisoning, is another well-known consequence of chronic lead exposure, with the most significant effects occurring in children. Lead exposure in children has been shown to impair neurological development, leading to cognitive deficits, behavioral issues, and lower IQ levels [62,63]. Lead also affects the hematological, renal, and reproductive systems, and chronic exposure has been linked to an increased risk of cardiovascular diseases in adults (World Health Organization) [64]. In both developed and developing countries, outdated infrastructure, such as lead-based plumbing systems, remains a significant source of exposure. For instance, the Flint water crisis in the United States, where elevated lead levels were found in the drinking water due to the corrosion of lead pipes, highlighted the continued risk of lead poisoning in modern-day infrastructure [65].

Similarly, Minamata disease in Japan serves as a stark example of the neurological effects of methylmercury poisoning. Between the 1950s and 1960s, thousands of individuals in Minamata Bay were exposed to high levels of methylmercury through the consumption of contaminated fish and shellfish, which had absorbed the mercury discharged from local industrial plants. The disease, which primarily affected the central nervous system, led to severe neurological impairments, including tremors, ataxia, and cognitive dysfunction [66]. Methylmercury is a potent neurotoxin, and its chronic exposure is linked to long-term cognitive and motor impairments, especially in fetuses and young children, who are more vulnerable to the neurotoxic effects [67].

Cadmium exposure, leading to the well-documented ‘itai-itai’ disease, serves as another example of heavy metal poisoning with severe health consequences. In the 1950s and 1960s, cadmium-contaminated water and rice in the Jinzu River basin in Japan led to kidney dysfunction, osteomalacia, and fractures in exposed populations. Chronic cadmium exposure causes renal tubular damage and inhibits calcium reabsorption, leading to weakened bones and increased fracture risk [68]. The disease is characterized by severe pain in the bones, hence the name “itai-itai” or “pain-pain” disease. Long-term cadmium exposure is also linked to an increased risk of cancer, particularly renal and prostate cancers, as well as cardiovascular diseases [69].

These diseases, although originating in specific regions and time periods, continue to be a global concern. They serve as a reminder that toxic metal exposure remains an important issue, both in terms of environmental contamination and public health. The persistence of these diseases underscores the need for stringent regulatory measures, public health interventions, and the ongoing monitoring of environmental pollutants to mitigate the health risks posed by toxic metals.

In addition to seafood, drinking water contamination by heavy metals further exacerbates human exposure. These metals leach into water supplies from industrial discharges, agricultural runoff, and urban pollution, contributing to additional health risks. The ingestion of contaminated water, especially with inorganic arsenic species, as well as lead ions (Pb^2+^), further exacerbates the risks posed by heavy metals. Chronic exposure, even at low concentrations, can disrupt metabolic processes, impair immune function, and contribute to developmental problems, particularly in children, pregnant women, and other vulnerable populations.

To protect against exposure to heavy metals in seafood and fruits, consumers should prioritize sourcing food from safe, certified origins. When purchasing fish, it is important to consider both the origin and safety standards. Sustainable fisheries and eco-friendly aquaculture systems typically comply with rigorous environmental regulations that limit the presence of harmful contaminants, including heavy metals and persistent organic pollutants. For example, fish from well-managed waters, such as wild-caught Alaskan salmon or farm-raised fish from eco-friendly operations, tend to have lower contamination levels. These sources are often subject to strict monitoring and testing for harmful substances, ensuring that the seafood is safe for consumption.

Similarly, when buying fruit, opting for certified organic or locally grown produce can minimize exposure to harmful chemicals, including pesticides and heavy metals that can be absorbed from contaminated soils. Fruits from regions with stringent agricultural standards, such as Europe or North America, are more likely to meet high food safety regulations and are less likely to carry harmful contaminants. Certifications such as GlobalGAP or USDA Organic ensure that best practices in farming and food safety are adhered to, which reduces the likelihood of contaminants in the food supply.

Consumers can also take additional precautions by thoroughly washing and peeling fruits and vegetables, which can help remove surface contaminants. This simple practice can further reduce the risk of exposure to harmful chemicals and make produce safer to consume.

Regions with the greatest heavy metal pollution are often characterized by industrial activity, mining, and agricultural practices that contribute to the accumulation of toxic metals in the environment [37,46]. Areas near large-scale mining operations, such as those in parts of Africa, South America, and Southeast Asia, are highly affected by the release of metals like elemental mercury (Hg^0^), Pb^2+^, and Cd^2+^ into surrounding ecosystems. In regions with intensive industrialization, such as Eastern Europe, China, and parts of India, heavy metal pollution is a significant concern due to emissions from manufacturing plants, smelting facilities, and coal-burning power stations. Additionally, agricultural areas where the use of contaminated water or pesticides is common, such as parts of South Asia, are prone to elevated levels of AsIII and AsV and Cd^2+^. Coastal regions with significant seafood production, including certain areas of the Mediterranean and East Asia, also experience CH_3_Hg^+^ contamination due to industrial discharges into the water. Urban centers worldwide, particularly in developing countries, may face heavy metal pollution from waste disposal, traffic emissions, and unregulated industrial activities. These regions, marked by a combination of industrial, agricultural, and environmental factors, often face serious public health risks related to heavy metal exposure.

Certain food products are more susceptible to contamination with heavy metals due to their ability to absorb or accumulate toxins from the environment. Seafood, particularly fish and shellfish, is often contaminated with MeHg (CH_3_Hg^+^), Cd^2+^, and Pb^2+^, as these metals accumulate in aquatic ecosystems and bio-magnify through the food chain. Leafy vegetables, such as spinach and lettuce, are prone to Cd^2+^ and Pb^2+^ contamination from polluted soils and water. Rice, particularly grown in areas with high levels of AsIII and AsV in the soil or irrigation water, can accumulate significant amounts of this toxic metal. Root vegetables like carrots and potatoes are also at risk, as they absorb heavy metals (Pb^2+^ and Cd^2+^) from the soil. Additionally, crops grown in contaminated environments, including fruits such as apples, pears, and grapes, may accumulate AsIII/AsV and Pb^2+^ from pesticides or polluted soil. Processed foods, especially those containing ingredients like rice or seafood, may carry higher levels of contaminants due to accumulation during production and packaging. Monitoring and regulation of these food sources are essential to minimize exposure to harmful metal species.

A dietary strategy for counteracting the toxic effects of heavy metals focuses on enhancing detoxification, reducing absorption, and promoting the repair of damage caused by these toxins [1]. Key components of such a strategy include the consumption of foods rich in antioxidants, such as vitamins C and E, which help neutralize reactive oxygen species (ROS) generated by heavy metal exposure. Increasing the intake of fiber from whole grains, fruits, and vegetables can aid in the elimination of heavy metals by binding to them in the digestive tract and reducing their absorption. Additionally, foods high in sulfur-containing compounds, like garlic, onions, and cruciferous vegetables, support the production of glutathione, a critical antioxidant involved in detoxification. The inclusion of selenium-rich foods, such as Brazil nuts and fish, also plays a role in neutralizing MeHg. Chelating agents, such as cilantro and pectin, found in certain fruits, have been suggested to support the body’s natural detoxification processes by binding to heavy metals and facilitating their excretion. Lastly, adequate hydration is essential to help flush out toxins through urine. This comprehensive dietary approach can help mitigate the toxic effects of heavy metals while promoting overall health and recovery.

#### 2.1.1. Mercury Contamination

Mercury (Hg) exists in multiple chemical forms, each exhibiting distinct toxicokinetic properties and toxicological effects. The toxicity of Hg is highly dependent on its chemical speciation, with elemental mercury (Hg^0^), inorganic mercury (Hg^2+^), and organic mercury compounds (e.g., methylmercury, MeHg) differing significantly in their bioavailability, distribution, metabolism, and excretion. The adverse health effects of mercury exposure range from acute toxicity to chronic bioaccumulation, with profound impacts on the nervous system, renal function, cardiovascular health, and immune response. While Hg^0^ is primarily hazardous through inhalation exposure, inorganic Hg^2+^ and MeHg pose serious risks upon ingestion, particularly through contaminated food and water sources [70,71].

Historically, the widespread industrial use of Hg led to numerous cases of occupational poisoning, particularly in Europe during the 19th century and in Korean industries such as thermometer and fluorescent lamp manufacturing. The implementation of stringent regulations since 2000 has significantly reduced Hg exposure through inhalation [72]. However, environmental contamination and dietary exposure remain major concerns. One of the most well-documented cases of mercury toxicity occurred in Minamata, Japan, in 1956, when industrial discharge led to the bioaccumulation of MeHg in marine organisms. This resulted in severe neurotoxic effects in humans, now recognized as Minamata disease [73].

Hg enters the human body through multiple routes, including the inhalation of atmospheric mercury vapor, ingestion of contaminated drinking water, and dietary intake of seafood and other Hg-laden food products [74]. Due to its lipophilicity, elemental Hg (Hg^0^) readily crosses alveolar membranes and is subsequently oxidized to Hg^2+^, which binds strongly to sulfhydryl (-SH) groups in proteins, leading to systemic accumulation [75]. Unlike elemental mercury, methylmercury (MeHg) is highly neurotoxic and bioaccumulates in aquatic food chains, posing a significant threat to human health. Importantly, exposure assessments should be based on specific Hg species, as different forms exhibit distinct toxicological profiles and metabolic pathways. The presence of Hg in food is particularly concerning, with exposure estimates necessary for evaluating risk levels. While the Hg content in food products is generally low, bioaccumulation in the food chain, especially in seafood, remains a significant exposure source. Moreover, mercury used in agriculture, such as seed treatment, can lead to residual contamination in cereal crops [76].

Marine environments serve as the primary reservoirs of mercury pollution, with significant anthropogenic inputs originating from industrial discharge, mining activities, fossil fuel combustion, and improper waste disposal [77]. Once introduced into aquatic ecosystems, Hg^2+^ undergoes biotransformation by microorganisms, leading to the production of MeHg, the most toxic and bioavailable form of mercury. This bioaccumulative property results in high Hg concentrations in predatory fish species, making dietary exposure a primary route for human intoxication. Notably, large marine predators such as sharks, swordfish, and tuna accumulate higher levels of MeHg compared to smaller fish species such as sardines and anchovies, which are generally considered safer alternatives [78,79]. Freshwater fish, in contrast, tend to have lower MeHg concentrations due to differences in aquatic biogeochemistry and food web dynamics [80].

The toxicokinetics of mercury species are critical to understanding their adverse health effects. MeHg readily crosses the blood–brain barrier and placental barrier, leading to neurodevelopmental toxicity, particularly in fetuses and young children. It binds to cysteine residues in proteins, mimicking methionine, which facilitates its transport into neural tissues. In contrast, inorganic mercury (Hg^2+^) preferentially accumulates in the kidneys, where it exerts nephrotoxic effects by disrupting cellular redox balance and inducing oxidative stress. Mechanistically, Hg^2+^ interacts with thiol (-SH) and selenol (-SeH) groups in proteins, impairing essential enzymatic functions and contributing to mitochondrial dysfunction, immune dysregulation, and endocrine disruption.

To assess human exposure to toxic mercury species, biomonitoring programs, such as those conducted by the Centers for Disease Control and Prevention (CDC) in the United States, provide critical data on population-wide exposure trends. These programs evaluate mercury levels in biological matrices, including blood, urine, and hair, to estimate cumulative exposure and potential health risks. Additionally, regulatory agencies such as the European Food Safety Authority (EFSA) and the U.S. Food and Drug Administration (FDA) have established permissible exposure limits for Hg species in food and water to mitigate public health risks. For instance, international trade regulations mandate that wheat intended for export must contain no more than 100 ppb of mercury, demonstrating the importance of strict monitoring and enforcement mechanisms in preventing heavy metal contamination.

Despite existing regulations, the link between chronic exposure to Hg species and the etiology of environmental diseases remains an active area of research. Epidemiological studies have associated prolonged exposure to MeHg with cognitive deficits, cardiovascular dysfunction, and immunotoxicity. Historical cases such as itai-itai disease (linked to cadmium poisoning in Japan) and plumbism (lead poisoning) underscore the long-term health implications of heavy metal exposure. Thus, further research is needed to elucidate the molecular mechanisms underlying Hg toxicity and to develop more effective public health interventions to minimize exposure risks.

Mercury compounds enter the human body through multiple exposure routes, including ingestion, inhalation, and dermal absorption, with the specific pathway and toxicity profile depending on the molecular form of mercury. Elemental mercury (Hg^0^) in vapor form is particularly hazardous due to its high absorption efficiency via the respiratory tract. Once inhaled, Hg^0^ readily diffuses across alveolar membranes and enters the bloodstream, where it exhibits a strong affinity for sulfhydryl (-SH) groups, binding preferentially to sulfur-containing amino acids and proteins. This interaction facilitates its systemic distribution, predominantly via erythrocytes and plasma proteins [81].

Due to its lipophilic nature, elemental mercury efficiently crosses both the blood–brain barrier and the placental barrier, leading to bioaccumulation in the fetal brain. Within the central nervous system (CNS), Hg^0^ undergoes oxidation to Hg^2+^, which has a high binding affinity for thiol-rich biomolecules, contributing to neurotoxicity through oxidative stress, the disruption of synaptic signaling, and mitochondrial dysfunction [82]. In addition to its neurotoxic effects, chronic exposure to elemental mercury vapors has been linked to peripheral neuropathies, renal impairment (due to preferential accumulation in proximal tubule cells), immunosuppression, endocrine dysregulation, and cardiotoxicity. Moreover, MeHg exposure has been associated with dermatological manifestations, including hypersensitivity reactions and various forms of dermatitis, further underscoring its systemic toxicity [83].

**Figure 3:**
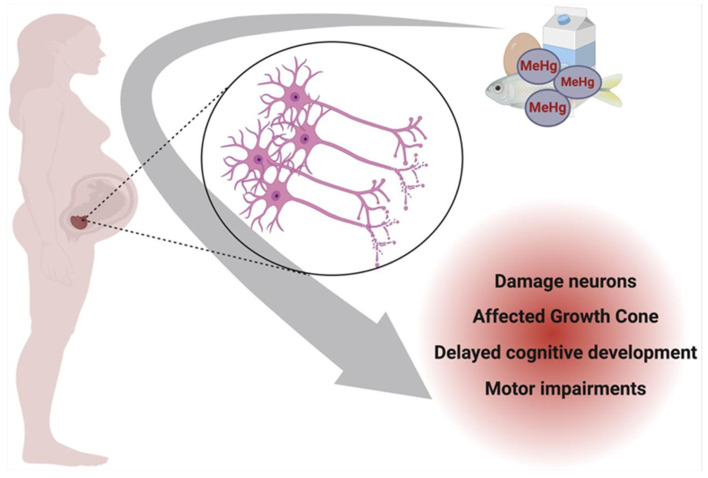
Prenatal neurotoxicity caused by Hg contamination in pregnant women. Created with BioRender.com (accessed on 25 October 2024).

Figure 3 illustrates the impact of prenatal exposure to specific mercury species on neuronal development. Methylmercury (MeHg), the predominant organic form of mercury in contaminated seafood, readily crosses the placenta after maternal ingestion, accumulating in the developing fetal brain. Within the nervous system, MeHg preferentially targets the growth cone of neurons, a critical structure for axonal elongation and synaptogenesis. In a physiologically normal growth cone, actin and tubulin neurofibrils maintain structural integrity and support neuronal outgrowth. However, MeHg disrupts cytoskeletal dynamics, impairing neurofibril organization and function, which can lead to developmental neurotoxicity and long-term cognitive deficits in the offspring [83].

A study conducted on 115 healthy individuals demonstrated a significant correlation between occupational exposure to elemental mercury (Hg^0^) vapors and an increased risk of cardiac dysfunction. Researchers concluded that chronic inhalation of Hg^0^ negatively impacts myocardial physiology and contributes to pathophysiological processes leading to left ventricular diastolic dysfunction [84].

Once absorbed, mercury species are transported through the bloodstream to various tissues, with their distribution dependent on their chemical form. Inorganic mercury (Hg^2+^) and MeHg exhibit distinct biodistribution patterns. While Hg^2+^ tends to accumulate in the liver, kidneys, pancreas, bladder, gastrointestinal mucosa, and, to a lesser extent, the brain, MeHg demonstrates a high affinity for neural tissue due to its ability to cross the blood–brain barrier. Within cells, mercury localizes to organelles such as mitochondria, microsomes, lysosomes, and nuclei, where it disrupts redox homeostasis and impairs cellular function. The primary excretory routes for Hg^2+^ include renal clearance via urine, hepatobiliary excretion via bile, and elimination through the intestinal mucosa, sweat glands, and salivary glands. In contrast, approximately 90% of ingested mercury is excreted through feces, while MeHg is predominantly eliminated via urinary excretion [85].

MeHg, primarily absorbed through dietary and inhalation exposure, exhibits nearly complete (approximately 90%) systemic absorption following ingestion [86]. Both in laboratory animals and humans, gut microbiota and macrophages facilitate the partial conversion of MeHg into inorganic Hg^2+^, which is characterized by prolonged retention in tissues. MeHg is rapidly distributed throughout the body, with the brain serving as its primary target due to significant accumulation. The estimated biological half-life of MeHg in humans who consume fish ranges between 39 and 70 days, with variations influenced by individual metabolic and excretory factors [87].

If dietary intake exceeds excretion rates, MeHg bioaccumulates, leading to increasing systemic burdens over time [88]. The toxicokinetics of MeHg are largely governed by its strong affinity for sulfhydryl (-SH) groups, facilitating its accumulation in the brain, muscle, and kidney tissues. MeHg readily penetrates the blood–brain barrier, where it undergoes demethylation, yielding inorganic Hg^2+^, which persists in neural tissue for years due to its extended half-life. Additionally, MeHg efficiently crosses both the blood–milk and placental barriers, posing significant risks to developing fetuses and nursing infants [89,90].

Cytogenetic studies using human lymphocyte cultures have demonstrated that exposure to methylmercury chloride (CH_3_HgCl), either alone or in combination with mercuric chloride (HgCl_2_), significantly increases the incidence of chromosomal aberrations [91]. CH_3_HgCl exposure is associated with a pronounced reduction in the mitotic index, indicative of cytotoxicity, along with heightened production of reactive oxygen species (ROS). Mercury’s high affinity for sulfhydryl groups in the mitotic spindle disrupts its function, resulting in chromosomal missegregation and conditions such as polyploidy or aneuploidy. Additionally, CH_3_HgCl has been reported to induce cytochrome c translocation from mitochondria into the cytosol of T-cells, initiating caspase cascade activation and leading to T-cell apoptosis [92].

Multiple mechanisms have been proposed to explain mercury-induced toxicity, including the upregulation of vascular endothelial growth factor (VEGF) expression in astrocytes, disturbances in intracellular calcium (Ca^2+^) homeostasis, interference with mitotic processes, immune system dysregulation, and the pathogenesis of neurodegenerative disorders [93,94]. The latency period for the onset of clinical manifestations can range from several months to years. Notably, systemic mercury concentrations do not always correlate with symptom severity, as some individuals may exhibit significant mercury burdens while remaining asymptomatic.

**Figure 4:**
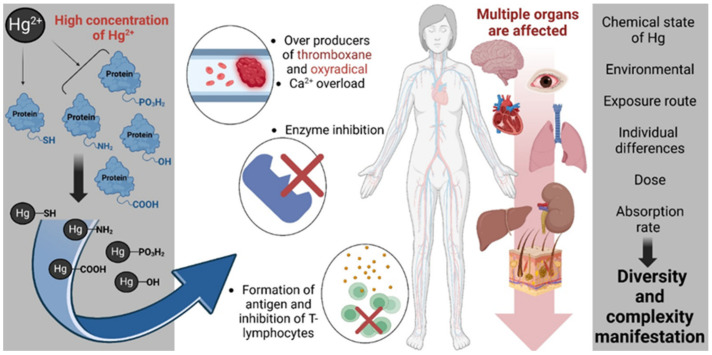
Mechanistic pathways of inorganic mercury (Hg^2+^) toxicity and systemic impact. Created with BioRender.com (accessed on 25 October 2024).

Figure 4 outlines the multifactorial biochemical and physiological mechanisms by which elevated concentrations of inorganic mercury (Hg^2+^) exert cytotoxic and immunotoxic effects [95]. Hg^2+^ demonstrates a high affinity for nucleophilic groups, forming covalent bonds with sulfhydryl (-SH), amine (-NH_2_), carboxyl (-COOH), hydroxyl (-OH), and phosphate (-PO_3_H_2_) moieties within proteins and cellular macromolecules. These interactions lead to the disruption of protein structure and function, resulting in the inhibition of critical enzymatic pathways (involved in cellular antioxidant defense, energy metabolism, and signal transduction). Notably, Hg^2+^ binding to -SH groups in glutathione reductase and glutathione peroxidase (GPx) impairs redox homeostasis, exacerbating oxidative stress. The inhibition of thioredoxin reductase, a selenoenzyme essential for DNA synthesis and cellular redox regulation, further amplifies oxidative damage. Additionally, Hg^2+^ interferes with Na^+^/K^+^-ATPase, compromising ion gradients and neuronal excitability, and disrupts mitochondrial enzymes such as cytochrome c oxidase, leading to impaired ATP production and mitochondrial dysfunction. Concurrently, Hg^2+^ exposure perturbs Ca^2+^ signaling, promoting intracellular Ca^2+^ overload and the generation of oxidative species such as oxyradicals and thromboxane, which contribute to vascular and neuronal dysfunction. Immunologically, Hg^2+^ may facilitate the formation of neoantigens, triggering autoimmunity and impairing T-lymphocyte function. These molecular and cellular disruptions culminate in multi-organ toxicity, with documented effects on the central nervous system, cardiovascular system, hepatic and renal tissues, pulmonary structures, and the gastrointestinal tract. The manifestation and severity of mercury-induced pathology are influenced by multiple variables including the chemical speciation of mercury, environmental context, route of exposure (e.g., inhalation, ingestion, dermal), absorbed dose, individual metabolic and genetic differences, and overall bioavailability. Collectively, these factors contribute to the high interindividual variability observed in mercury toxicity profiles.

The cardiotoxic effects of mercury have been well-documented in in vitro models, animal studies, and human epidemiological investigations [96]. A significant correlation has been identified between dietary mercury exposure and increased blood pressure, with prolonged exposure contributing to the development of hypertension. Furthermore, mercury-induced autonomic dysfunction is evidenced by reduced heart rate variability, a marker of impaired cardiovascular regulation. Mercury exposure has also been associated with an increased risk of atherosclerosis, myocardial infarction, and coronary artery disease [96]. The concentration of mercury detected in hair has been found to be predictive of oxidized low-density lipoprotein (oxLDL) levels, a key biomarker of atherosclerosis and cardiovascular pathology [96]. Mercury exacerbates LDL oxidation by inactivating paraoxonase-1 (PON-1), a Ca^2+^-dependent extracellular enzyme with antioxidant and anti-atherogenic properties. PON-1 primarily inhibits the oxidation of LDL and high-density lipoprotein (HDL) cholesterol, and its inactivation by mercury contributes to oxidative stress and vascular inflammation.

Mercury toxicity significantly enhances the production of reactive oxygen species (ROS). By binding to -SH groups in cellular proteins and forming selenium–mercury (Se–Hg) complexes, mercury disrupts the function of key antioxidant enzymes, including glutathione peroxidase, catalase (CAT), and SOD. This disruption disturbs redox homeostasis, creating a sustained pro-oxidative state that contributes to cardiovascular disease and systemic oxidative damage [96].

At the mitochondrial level, mercury induces phosphatidylserine translocation within the mitochondrial membrane, leading to alterations in membrane integrity, the disruption of mitochondrial membrane potential, and the activation of apoptotic pathways [96].

The cardiotoxic effects of mercury manifest through a spectrum of pathological outcomes, including hypertension, myocardial infarction, reduced heart rate variability due to autonomic dysfunction, increased carotid intima-media thickness (IMT), carotid artery obstruction, atherosclerosis, arrhythmias, and progressive renal dysfunction culminating in kidney failure. In severe cases, mercury-induced cardiovascular toxicity may lead to sudden cardiac death.

To mitigate cardiovascular complications associated with mercury exposure, researchers recommend dietary supplementation with omega-3 polyunsaturated fatty acids (PUFAs), preferably sourced from fish oil or plant-based alternatives, as well as selenium supplementation to counteract mercury-induced oxidative stress and enzymatic inhibition [96].

The consumption of mercury-contaminated food represents a significant public health concern. The United States Environmental Protection Agency (EPA) and the National Academy of Sciences have established recommended exposure limits to minimize health risks. Specifically, total blood mercury concentrations should remain below 5.0 μg/L, while hair mercury levels should not exceed 1.0 μg/g. These thresholds correspond to a reference dose (RfD) of 0.1 μg/kg body weight per day, which is considered the maximum tolerable intake to prevent adverse health effects [97].

Mercury exposure remains a significant global health concern due to its neurotoxic effects, bioaccumulation in the food chain, and persistence in the environment. Regulatory bodies have established strict limits to reduce human exposure. EFSA sets a tolerable weekly intake (TWI) of 1.3 µg/kg body weight for methylmercury, the most toxic form. The World Health Organization (WHO) and the United States Environmental Protection Agency (EPA) regulate mercury levels in drinking water at 6 µg/L and 2 µg/L, respectively. The Codex Alimentarius establishes maximum levels in fish and seafood due to their role as primary dietary sources. Biomonitoring studies highlight the need for continuous monitoring, particularly in populations with high fish consumption, to prevent neurodevelopmental and systemic toxic effects [96,97].

Table 2 highlights the levels of Hg contamination in various food products across multiple countries, with an emphasis on the diverse sources of Hg exposure through dietary intake.

#### 2.1.2. Cadmium Contamination

Cd is commonly used as an anti-corrosion agent, applied to metal containers to prevent rusting. It is also found in batteries, plastics, paints, and electroplating processes. During metal production and the burning of fossil fuels, Cd is released into the atmosphere. Another significant source of Cd release is the use of phosphate-based fertilizers and sewage sludge [106].

Rather than the vague statement, “Cd levels in food are generally low,” it is more accurate to provide specific concentration ranges. For example, Cd concentrations in food typically range from 0.01 to 0.2 mg/kg in staple crops such as cereals and vegetables [107]. Additionally, Cd’s gastrointestinal absorption is limited to about 5–10% of the ingested dose, depending on dietary factors and the individual’s nutritional status. This bioavailability, however, can vary significantly in specific populations, which should be addressed when evaluating the health risks associated with Cd exposure.

Despite relatively low concentrations in food, daily exposure, combined with its prolonged biological half-life (approximately 25–30 years), can lead to significant accumulation in the body over time. The ionic species of concern, primarily Cd^2+^, can be absorbed into the bloodstream and accumulate in tissues. As a ubiquitous environmental contaminant, Cd^2+^ poses considerable health risks. Long-term exposure, even at low levels, can lead to renal tubular dysfunction and bone toxicity, and it has been linked to various forms of cancer, particularly hormone-dependent cancers like breast cancer [107,108,109]. Recent evidence also suggests that Cd^2+^ exposure may adversely affect neurodevelopment, making it a concern for pregnant women, infants, and children [109].

Cd^2+^ is absorbed by plants through their root systems, making cereals, vegetables, and other plant-based foods the primary dietary sources of Cd. Certain population groups are more vulnerable to Cd^2+^ exposure, including infants, vegetarians, smokers, women of childbearing age with low iron status (which enhances the gastrointestinal absorption of Cd^2+^), and individuals with pre-existing renal dysfunction [108]. Cd^2+^ tends to accumulate in plant leaves, with tobacco being of particular concern. Smoking tobacco leaves represents a major source of Cd^2+^ exposure for both active smokers and non-smokers exposed to secondhand smoke [110].

Marine organisms, such as oysters, mussels, and other invertebrates, can contain substantial concentrations of Cd^2+^, especially in areas of high pollution. This makes seafood a significant concern for populations that consume these organisms in large quantities [111,112].

Dietary Cd^2+^ intake is largely influenced by food consumption patterns and the Cd^2+^ concentrations present in consumed products. While foods with high Cd^2+^ concentrations, such as oilseeds, herbs, and certain seafood, contribute minimally to daily Cd^2+^ intake, staples like cereals, vegetables, rice, root crops, and potatoes, which generally contain moderate Cd^2+^ concentrations (≤0.03 mg/kg), are the primary contributors to dietary Cd^2+^ exposure [112].

**Figure 5:**
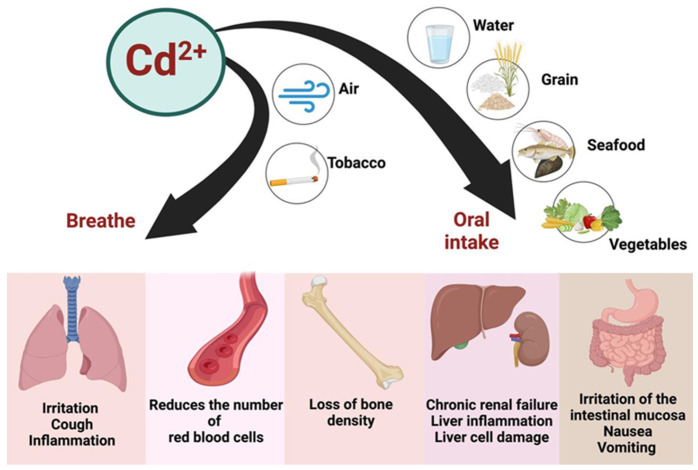
The sources of contamination, major pathways of Cd entry into the human body, and target organs where detrimental effects are exerted. Created with BioRender.com (accessed on 25 October 2024).

Exposure to Cd^2+^ occurs not only through the ingestion of contaminated food and water but also via inhalation and smoking (Figure 5). Once absorbed, Cd^2+^ accumulates in the body, with a biological half-life of approximately 25–30 years [113]. The liver and kidneys are particularly vulnerable to the toxic effects of Cd^2+^, but its impact on the cardiovascular system also warrants attention [6]. Cd^2+^ exposure has been linked to oxidative stress, endothelial dysfunction, and increased vascular permeability, which may contribute to the development of cardiovascular diseases [6].

Cd is primarily absorbed through the respiratory tract and, to a lesser extent, through the gastrointestinal tract. Dermal absorption is relatively rare or negligible. Once inside the body, Cd^2+^ is transported via the bloodstream, bound to erythrocytes and albumin, and subsequently accumulates in organs such as the kidneys, liver, pancreas, and intestines [114,115]. Cd^2+^ is excreted slowly from the body, predominantly via the kidneys, urine, saliva, and breast milk during lactation. Chronic exposure to Cd^2+^ in humans is associated with a broad spectrum of adverse health effects, including renal and hepatic dysfunction, pulmonary edema, testicular damage, osteomalacia, and impairments to the adrenal glands and hematopoietic system [116].

Among the various health impacts of Cd^2+^ exposure, itai-itai disease stands out as one of the most extensively documented cases. This debilitating condition was first identified in Japan, where it was linked to the consumption of fish and shellfish contaminated with elevated levels of Cd^2+^. Affected individuals experienced severe bone and joint pain, spontaneous bone fractures, and other serious health complications, often resulting in death [114,117]. Cd^2+^-induced osteomalacia is primarily linked to insufficient vitamin D intake combined with elevated dietary Cd^2+^ exposure [118]. Additionally, Cd^2+^ exhibits significant mutagenic and carcinogenic effects, making it one of the most potent metallic carcinogens—classified as a Group 1 carcinogen by IARC [119]. Its toxicity impacts all organ systems, posing a substantial threat to human health [107].

Existing reports in the scientific literature have identified significant associations between Cd^2+^ exposure and various neoplastic diseases, including breast, lung, prostate, nasopharyngeal, pancreatic, and kidney cancers, as well as osteoporosis [107]. Cd^2+^ exposure can impair the immune system by inducing oxidative stress and triggering epigenetic changes at the cellular level, as demonstrated by both in vivo and in vitro studies. These alterations contribute to pathogenic risks and the development of various cancers. Cd^2+^ induces a range of epigenetic modifications, including chemical alterations to DNA and histones, which disrupt chromatin structure without altering the nucleotide sequence of DNA [120]. Its tumorigenic mechanisms are complex and multifactorial, involving DNA repair inhibition, apoptosis stimulation, oxidative stress induction, and the activation of aberrant gene expression [121]. The presence and accumulation of Cd^2+^ in the body have been associated with lung, renal, and prostate cancers [122,123].

Cadmium’s genotoxic potential is largely mediated through the generation of ROS, which play a central role in most genotoxic events observed in cells exposed to Cd^2+^. This results in hallmark features of oxidative stress, including mutagenic DNA lesions, such as 8-oxo-7,8-dihydro-2′-deoxyguanosine (8-oxodG), which has been detected in the DNA of human lymphoblastoid cells [124].

Cd^2+^ directly impacts the heart and vascular structures essential for the overall functionality of the cardiovascular system. Cd^2+^ disrupts vascular endothelium integrity, leading to endothelial dysfunction, a precursor to atherosclerosis. Studies have also demonstrated associations between Cd^2+^ exposure markers (blood and urine levels) and an increased prevalence of coronary artery disease, stroke, peripheral arterial disease (PAD), and atherogenic lipid profile changes. Urinary Cd^2+^ levels have been correlated with the severity of PAD in patients with coronary artery disease, with higher levels indicating greater disease severity and significant vascular complications, such as ulcers and gangrene. Similarly, elevated urinary Cd^2+^ concentrations have been linked to a higher incidence of heart failure, as observed in the Strong Heart, Hortega, and Danish Diet, Cancer, and Health studies, particularly among men [6].

Cadmium poisoning disrupts normal physiological functions by interfering with key enzymatic processes and cellular functions. Upon absorption, Cd^2+^ primarily accumulates in the kidneys and liver, where it binds to proteins such as metallothionein and induces oxidative stress through the generation of ROS. This oxidative stress damages cellular structures, including lipids, proteins, and DNA, leading to cell death and tissue dysfunction. Cd^2+^ inhibits the activity of critical enzymes, such as glutathione-S-transferase, which is involved in detoxification processes, and SOD, an antioxidant enzyme, further exacerbating oxidative damage. Additionally, Cd^2+^ disrupts the function of enzymes in energy metabolism, including those involved in the citric acid cycle, thereby impairing ATP production and mitochondrial function. It also interferes with calcium signaling by affecting calcium-dependent enzymes, contributing to renal and bone damage. Chronic exposure to Cd^2+^ can lead to kidney damage, bone demineralization, and an increased risk of cancer due to its interference with DNA repair enzymes and disruption of cellular repair mechanisms. These combined effects on enzymatic pathways and cellular functions highlight the systemic toxicity induced by Cd^2+^.

Cd exposure is a critical public health concern due to its bioaccumulative nature and toxic effects on the kidneys, bones, and cardiovascular system. Regulatory agencies have established strict limits to minimize risk. EFSA sets a tolerable weekly intake (TWI) of 2.5 µg/kg body weight, while the WHO and the United States FDA regulate Cd^2+^ levels in drinking water at 3 µg/L. The Codex Alimentarius provides maximum permissible levels for Cd^2+^ in foodstuffs such as cereals, vegetables, and seafood. Biomonitoring data indicate that long-term exposure, primarily from diet and tobacco smoke, necessitates ongoing surveillance and stricter regulatory enforcement to mitigate health risks [107,112,117].

Table 3 systematically presents the Cd contamination levels in food products from various countries, offering a detailed comparison of the levels found across different food categories and regions.

#### 2.1.3. Lead Contamination

Pb is a non-essential and highly toxic element that adversely affects multiple biological systems in humans. Major sources of exposure include occupational settings such as leaded gasoline production, boat manufacturing, lead-based paints, and battery recycling. Additionally, ingestion through contaminated food and drinking water represents a significant exposure route. Once absorbed, approximately 90% of Pb^2+^ accumulates in bone tissue, where it replaces Ca^2+^, leading to reduced bone mineral density and long-term retention. Chronic Pb^2+^ exposure is particularly detrimental to the central nervous system (CNS), impairing memory and motor function, especially in children [146].

In circulation, Pb^2+^ is primarily transported by erythrocytes, binding to their surface before distributing to various tissues and organs. Bone tissue serves as the primary long-term storage site, with Pb^2+^ initially deposited in a colloidal form and later transforming into crystalline structures. The concentration of Pb^2+^ in bone depends on cumulative exposure duration, whereas its levels in soft tissues remain relatively stable over time [147].

The neurotoxic effects of Pb^2+^ can be categorized into morphological and pharmacological disruptions. Morphologically, Pb^2+^ interferes with neuronal development from the prenatal period through childhood by impairing neuronal migration, differentiation, and synapse formation due to reduced sialic acid production. It also accelerates premature glial cell differentiation [148]. Pharmacologically, Pb^2+^ disrupts CNS function by displacing Ca^2+^ and Zn^2+^, leading to the aberrant activation of calmodulin-dependent pathways. This interference alters neurotransmitter release, impairing GABAergic, dopaminergic, and cholinergic systems while inhibiting N-methyl-D-aspartate (NMDA) ion channels. Pb^2+^ also disrupts intracellular Ca^2+^ homeostasis, triggering reactive oxygen species (ROS) production, mitochondrial dysfunction, and apoptosis via mitochondrial permeability transition pore formation [149,150].

Clinically, Pb^2+^ poisoning, or saturnism, presents with varying severity. Mild exposure leads to reduced nerve impulse conduction velocity [151], while high dietary intake results in peripheral nerve damage correlated with anemia severity. Excessive Pb^2+^ ingestion induces gastrointestinal inflammation. Historical cases of Pb^2+^ poisoning have been linked to the consumption of alcoholic beverages stored in ceramic containers with Pb-based glazes or improperly distilled liquors. In food sources, Pb^2+^ concentrations are generally low in muscle and organ meats, except for kidneys, where levels may range from 0.1 to 2.75 mg/kg, and livers, which may contain up to 0.40 mg/kg. Meat typically contains less than 0.05 mg/kg of Pb^2+^, classifying it as a low-lead-content food [152,153,154].

Additional sources of Pb^2+^ exposure include low-quality jewelry and accessories, often manufactured with substandard materials. A preliminary study on jewelry available in Nigeria revealed that 12% of tested items exceeded EU safety limits for Pb^2+^, 63% for Cd^2+^, 42% for chromium (Cr^3+^), and 62% for nickel (Ni^2+^) [155]. This concern is exacerbated by the rise of e-commerce platforms facilitating the sale of low-quality accessories, increasing the risk of toxic metal exposure [156,157].

At the biochemical level, Pb^2+^ toxicity induces systemic disruptions primarily by interfering with enzymatic processes. Pb^2+^ inhibits enzymes critical for heme biosynthesis, including δ-aminolevulinic acid dehydratase (ALAD) and ferrochelatase, leading to the accumulation of toxic precursors such as δ-aminolevulinic acid (ALA) and protoporphyrin IX. This disruption results in anemia and impaired oxygen transport. Pb^2+^ also inhibits ATPases, disrupting ion gradients and mitochondrial function, contributing to cell death. By interfering with antioxidant systems such as glutathione (GSH) and SOD, Pb^2+^ promotes oxidative stress, leading to lipid, protein, and DNA damage. Furthermore, Pb^2+^ affects neurotransmitter release by disrupting calcium-dependent signaling, exacerbating neurotoxicity, particularly in developing nervous systems. Chronic exposure impairs cytochrome P450 enzyme activity, reducing detoxification efficiency [6].

Regulatory agencies have established stringent Pb^2+^ exposure limits to mitigate health risks. The U.S. Food and Drug Administration enforces a maximum Pb^2+^ concentration of 5 parts per billion (ppb) in bottled water under the Federal Food, Drug, and Cosmetic Act. In contrast, the U.S. Environmental Protection Agency permits a higher threshold of 15 ppb for Pb^2+^ in drinking water, accounting for potential contamination from aging plumbing infrastructure [158,159]. To further protect vulnerable populations, specific limits apply to children’s food products, with maximum allowable Pb^2+^ concentrations set at 0.1 ppm for candies and 50 ppb for fruit juices [160].

Given Pb^2+^’s well-documented toxicity, continuous biomonitoring is critical. The Centers for Disease Control and Prevention (CDC) conduct regular surveillance to assess human Pb^2+^ exposure, utilizing biomonitoring data as a cornerstone for public health interventions. Effective risk mitigation strategies include stricter environmental policies, improved food safety regulations, and public education initiatives to reduce Pb^2+^ exposure and its associated health risks. Addressing these issues comprehensively is essential to minimizing Pb^2+^-related health and environmental hazards.

Table 4 provides a comprehensive overview of Pb contamination levels in food products across various countries, highlighting the global variability in Pb exposure through diet.

#### 2.1.4. Arsenic Contamination

Historically, As was widely used as both a poison and a medicinal agent before the advent of antibiotics. Today, inorganic arsenic species, particularly arsenite (AsIII) and arsenate (AsV), are recognized as highly toxic and carcinogenic, with profound adverse effects on multiple biological systems [169].

The primary sources of arsenic exposure include contaminated water, soil, food, and air. The ingestion of water contaminated with inorganic arsenic leads to nearly 100% absorption through aquaporin channels in the gastrointestinal tract, whereas arsenic from contaminated food is absorbed at lower rates, typically less than 50%. Inhalation of airborne arsenic compounds results in absorption levels below 50%, while dermal exposure contributes minimally, with less than 5% absorption [6].

Following absorption, inorganic arsenic undergoes hepatic biomethylation, yielding monomethylarsonic acid (MMA) and dimethylarsinic acid (DMA). This metabolic process is influenced by genetic polymorphisms affecting methylation efficiency [169,170]. Unlike other heavy metals, AsIII and AsV, along with their methylated metabolites, are primarily excreted via urine, and the cessation of exposure significantly reduces their systemic toxicity [6].

Despite its elimination, arsenic exposure induces physiological disturbances that can lead to systemic pathological conditions. At the vascular endothelial level, AsIII upregulates the expression of cellular adhesion molecules, disrupting intracellular signaling and promoting pro-atherogenic mechanisms. These effects include increased vascular permeability, enhanced cellular adhesion, oxidative stress, and localized inflammatory responses. Additionally, arsenic disrupts lipid metabolism by promoting lipid retention within macrophages, leading to foam cell formation and contributing to atherosclerotic plaque development [6].

Arsenic exerts cardiotoxic effects by disrupting intracellular Ca^2+^ homeostasis in myocardial tissue. This occurs through the downregulation of the cardiac potassium channel associated with the human ether-à-go-go gene (hERG), which increases the risk of QT interval prolongation and predisposes individuals to torsades de pointes, a form of life-threatening ventricular tachycardia [171]. While this effect is well documented in arsenic trioxide therapy for acute promyelocytic leukemia, chronic low-to-moderate exposure to inorganic arsenic has been associated with similar cardiac outcomes [172].

At the cellular level, arsenic exerts its toxic effects by interfering with critical enzymatic functions. AsIII mimics phosphate groups and binds to sulfhydryl (-SH) groups in proteins, leading to the inhibition of essential enzymes. It disrupts cellular respiration by inhibiting pyruvate dehydrogenase and α-ketoglutarate dehydrogenase, enzymes essential for the citric acid cycle. Arsenic also impairs DNA synthesis and repair by inhibiting methionine synthase, leading to genomic instability. Furthermore, AsIII suppresses the activity of antioxidant enzymes such as GPx, exacerbating oxidative stress and cellular damage. Chronic exposure inhibits cytochrome P450 enzymes, impairing detoxification processes, and disrupts apoptosis regulation by interfering with protein kinase signaling pathways. These disruptions contribute to organ toxicity, immune dysfunction, and an elevated risk of carcinogenesis.

Chronic exposure to inorganic arsenic species poses severe health risks across multiple organ systems, leading to progressive pathological changes depending on the site of impact [173]:

- Integumentary system: hyperpigmentation, leukoderma, and cutaneous lesions, including keratosis and skin cancer.
- Urinary system: degeneration of proximal renal tubules, cortical and papillary necrosis, and increased risk of urothelial carcinoma.
- Nervous system: encephalopathy affecting the central nervous system, and peripheral neuropathy characterized by sensory and motor deficits.
- Liver: hepatomegaly, cirrhosis, and disrupted heme metabolism, contributing to hepatotoxicity.
- Endocrine system: increased risk of diabetes mellitus due to arsenic-induced pancreatic β-cell dysfunction.
- Hematopoietic system: suppression of bone marrow function, leading to anemia and leukopenia.

Given the significant global health concerns associated with arsenic exposure, it is imperative to address human exposure to toxic metal species in drinking water, food, and the environment. Arsenic exposure poses a significant global health risk, with regulatory agencies establishing strict safety limits. The European Union sets a maximum contaminant level of 10 µg/L for arsenic in drinking water, aligning with the United States Environmental Protection Agency and FDA standards. Dietary exposure is also regulated, with the European Food Safety Authority identifying a benchmark dose lower confidence limit (BMDL01) of 0.3–8 µg/kg body weight per day, linked to increased cancer risks. Biomonitoring studies reveal widespread exposure, particularly in regions with high natural arsenic levels, underscoring the need for continuous surveillance and risk mitigation strategies [169,170,171,172,173].

Table 5 outlines As contamination levels in various food products across different countries, with an emphasis on the diversity of As exposure through dietary sources.

#### 2.1.5. Tin Contamination

Tin (Sn) is widely utilized in both industry and agriculture [177]. It is employed in tinning steel sheets used for manufacturing food cans and as an alloy for soldering copper containers designated for canned goods. Additionally, Sn is used in producing household items. The presence of Sn in food products primarily results from residual accumulation in raw materials due to the application of fungicides containing Sn, as well as from the corrosion of containers used for food packaging [178].

Elemental Sn, in its metallic form, is not toxic to animals or humans [179]. However, its inorganic species, particularly Sn(II) (stannous) and Sn(IV) (stannic) compounds, exhibit varying degrees of toxicity. Tin(II) chloride (SnCl_2_) and Tin(IV) chloride (SnCl_4_), which are by-products of container corrosion in high sodium chloride (NaCl) environments, are considered toxic. While human toxicity remains relatively low, lethal effects are only observed at doses exceeding 5000 mg. The liver, kidneys, and spleen are the primary organs for Sn storage, but significant accumulation occurs only following substantial ingestion [179].

Tin toxicity disrupts physiological and enzymatic functions through multiple mechanisms. Specifically, inorganic Sn species interfere with key metabolic pathways by binding to sulfhydryl (-SH) groups in enzymes, leading to their inhibition. This impairment affects enzymes such as pyruvate dehydrogenase and α-ketoglutarate dehydrogenase, both essential for mitochondrial function and ATP production. Moreover, Sn exposure generates reactive oxygen species (ROS), inducing oxidative stress that damages cellular lipids, proteins, and DNA. Antioxidant defense systems, including SOD and CAT, become overwhelmed, exacerbating oxidative damage. In the nervous system, inorganic Sn species disrupt neurotransmitter synthesis and degradation, impairing synaptic signaling and contributing to neurotoxicity. Additionally, Sn accumulates in the liver and kidneys, inhibiting detoxification enzymes such as cytochrome P450 and glutathione S-transferase, leading to systemic toxicity and organ dysfunction. These combined effects highlight the broad metabolic and enzymatic disruptions caused by tin poisoning.

The following section outlines the effects of inhalation exposure to both inorganic and organic Sn compounds, emphasizing gaps in research and documented adverse effects [179]:

- Cardiovascular effects: Research indicates that exposure to Sn vapors negatively impacts myocardial function, particularly by inducing left ventricular diastolic dysfunction [180].
- Respiratory effects: Exposure to inorganic Sn compounds has been associated with stannosis, a benign pneumoconiosis that does not impair lung function. However, organotin compounds, such as tributyltin (TBT) and dibutyltin (DBT), can cause severe respiratory distress, sometimes necessitating artificial ventilation, though these effects typically do not result in lasting respiratory complications [181,182].
- Gastrointestinal effects: Limited data exist regarding the effects of organotin compounds, but reports indicate nausea and abdominal pain following exposure [178,179,183].
- Ocular effects: A study reports that tributyltin chloride exposure in rats resulted in periorbital edema and conjunctivitis [184].
- Immunological and lympho-reticular effects: Limited data suggest that inorganic Sn exposure may lead to lymph node atrophy in rats [185,186].
- Neurological effects: Organotin compounds have been linked to neurotoxicity, behavioral changes, headaches, and memory deterioration in humans [187].

Given the significant gap in research concerning Sn exposure compared to other heavy metals (e.g., Hg, Cd, Pb), there is a critical need for studies identifying the most contaminated food products, assessing risks associated with both chronic low-dose exposure and acute high-dose exposure (e.g., industrial accidents or errors in food can manufacturing). Furthermore, future research should prioritize developing cost-effective, feasible, and easily implementable strategies to mitigate the spread of toxic tin compounds and their impact on various human organ systems.

Tin exposure, particularly from inorganic and organotin compounds, raises concerns due to its potential toxicological effects. Regulatory agencies have established safety thresholds to minimize health risks. The EFSA sets a tolerable daily intake (TDI) of 0.6 mg/kg body weight for inorganic tin, while the Codex Alimentarius Commission limits tin in canned beverages to 150 mg/kg and in other canned foods to 250 mg/kg. The United States FDA also regulates tin levels in food packaging materials. Biomonitoring data indicate that dietary intake is the primary exposure route, necessitating stringent controls and continuous assessment to prevent adverse health effects [179,180,181,182,183,184,185,186,187].

Table 6 presents Sn levels in various food products from different countries, highlighting the contamination observed in preserved foods, fish, and other consumables.

### 2.2. Strategies for Risk Reduction

In light of scientific evidence, it is imperative to implement strategies aimed at reducing the risk of contamination of both food products and the environment with heavy metals. These strategies are critical for safeguarding public health and ensuring food safety. Approaches to mitigate the risk of food contamination with toxic heavy metal species, such as Pb^2+^, Cd^2+^, arsenic (AsIII and AsV), Hg^2+^, and others, are essential. These strategies include contamination prevention, mitigation techniques, and remediation methods, such as phytoremediation [193]. Phytoremediation, a bioremediation process utilizing plants, can effectively purify soil, water, and air, removing both organic and inorganic pollutants, including toxic metals, from various environments [194]. To successfully implement these strategies, a collaborative effort among researchers, farmers, the food industry, and regulatory authorities is required. This coordination ensures the adoption of the most effective risk management practices. Continuous research is essential to the development and optimization of these strategies to protect both human health and the environment, necessitating joint efforts to achieve progress in this area.

Biomonitoring data from the Centers for Disease Control and Prevention (CDC), specifically through the National Health and Nutrition Examination Survey (NHANES), provide critical insights into human exposure to toxic metals or metalloids. According to NHANES data, median blood levels for key toxic metals in the U.S. population are approximately as follows [195]:

- As: The total urinary arsenic median level is 8.4 µg/L, though this varies depending on seafood consumption.
- Cd: The median blood cadmium concentration is 0.3 µg/L, with higher levels in smokers due to tobacco-related exposure.
- Hg: The median blood mercury level is 0.86 µg/L, though this is significantly higher in populations with frequent fish consumption.
- Pb: The median blood lead level is 0.85 µg/dL, reflecting a decline due to regulatory efforts but still posing risks in vulnerable populations.

Biomonitoring data, especially from reliable sources such as the Centers for Disease Control and Prevention (CDC), offer essential insights into the levels of toxic metals in the general population, facilitating risk assessments and public health initiatives. The CDC’s National Health and Nutrition Examination Survey (NHANES) provides an extensive dataset that monitors the exposure of Americans to heavy metals like Pb^2+^, Cd^2+^, As, and Hg (Hg^0^, Hg^2+^, and MeHg). According to the most recent NHANES data, the median blood lead concentration in U.S. adults has declined significantly from 1999 to 2018, but about 2.5% of children aged 1–5 years still have blood lead levels above the reference value of 5 µg/dL, with higher risks observed in children living in older housing or in low-income areas. Cd^2+^ exposure, primarily through smoking and dietary sources, remains a concern, with NHANES indicating a median blood Cd^2+^ concentration of 0.3 µg/L in the adult population, which is notably higher among smokers (0.8 µg/L). This exposure can contribute to kidney damage and increase the risk of cardiovascular disease. Mercury exposure is also tracked, with the median blood mercury level at 0.86 µg/L, though this varies widely depending on fish consumption patterns. For instance, mercury concentrations are higher in individuals who frequently consume large predatory fish such as tuna or swordfish [196]. Additionally, arsenic levels in the population, primarily from drinking water and rice consumption, are being closely monitored, with data showing that about 10% of individuals had detectable levels of inorganic arsenic in urine, which is a strong indicator of long-term exposure. These data are essential for identifying high-risk populations, informing policy changes, and improving prevention strategies aimed at reducing exposure to toxic metals. Furthermore, this biomonitoring information supports the effectiveness of regulations, such as those imposed by the EPA and FDA, that limit exposure to harmful levels of heavy metals in food and drinking water [197].

While exposure to heavy metals may seem unavoidable due to their presence in the environment, food, and water, effective strategies do exist to significantly reduce the risk of accumulation in the human body [198,199,200]. To minimize exposure, we propose several approaches targeting specific ionic species involved in heavy metal toxicity:

- Careful selection of food products: Some species of fish and seafood, such as large predatory fish, tend to accumulate higher levels of toxic metals or metalloids like mercury (Hg^2+^) and arsenic (AsIII and AsV). Fish with lower mercury levels, such as salmon, tilapia, sardines, cod, sole, trout, and herring, should be preferred [201]. To minimize exposure to a single contaminant, consuming a diverse range of fruits and vegetables is recommended, ideally opting for organic produce when possible.
- Water filtration: In areas where water quality falls below recommended standards, the use of certified filtration systems capable of removing metal or metalloid ions like Pb^2+^, Cd^2+^, and AsIII is essential.
- Soil testing and remediation: Regular testing of agricultural soil for heavy metal contamination, followed by remediation strategies such as phytoremediation or soil amendments to immobilize metal or metalloid ions like Pb^2+^, Cd^2+^, and AsIII, is essential [202].
- Crop selection: Growing crops that are less prone to accumulating heavy metals, especially in areas with known contamination risks, can minimize exposure to toxic species such as Cd^2+^ and Pb^2+^.
- Regulation of industrial emissions: Strict guidelines must be enforced to regulate industrial emissions of heavy metals, including Pb^2+^, Cd^2+^, and Hg^2+^, from factories, mining operations, and power plants, to reduce atmospheric deposition into the environment.
- Proper waste management: Proper disposal and recycling of electronic waste and batteries, which often contain high levels of Pb^2+^ and Hg^2+^, must be implemented to prevent leaching into soil and water [203,204].
- Green buffer zones: Establishing green buffer zones around industrial areas can limit the drift of heavy metal ions, such as Pb^2+^, into agricultural fields.
- Public education campaigns: Raising awareness about the sources and risks of heavy metal exposure, particularly the toxicity of ions such as Pb^2+^, Cd^2+^, AsIII, and Hg^2+^, is essential to mitigating health risks.
- Certified food products: Encouraging consumers to select certified organic or heavy metal-tested food products can help reduce exposure to harmful metals or metalloids like AsIII, Pb^2+^, and Cd^2+^.
- Improving indoor air quality: The use of air purifiers can minimize inhalation of heavy metal particles, especially in urban or industrial areas, which may contain Pb^2+^ and Cd^2+^.
- Limiting cosmetic and traditional medicine use: Certain cosmetics and traditional medicines may contain toxic metals like Pb^2+^ and Hg^2+^, which can be absorbed through the skin or ingested, increasing the burden of these metals in the human body [205,206].
- Food monitoring programs: Strengthening government-led monitoring programs for heavy metal contamination in food products, especially monitoring for Pb^2+^, Cd^2+^, AsIII, and Hg^2+^, can help track and mitigate risks to public health.
- Environmental cleanup initiatives: National and regional programs focused on cleaning contaminated rivers, lakes, and industrial waste sites are necessary to reduce exposure to toxic metal species such as Pb^2+^ and Cd^2+^.
- Bioremediation technologies: Exploring microbial and enzymatic solutions for detoxifying environments contaminated with heavy metals like AsIII, Hg^2+^, and Pb^2+^ is a promising strategy.
- Food storage recommendations: Avoiding the use of ceramic or colored glassware that may contain Pb^2+^ and opting for glass or stainless-steel containers for food storage can help limit exposure to Pb^2+^.
- Maintaining a balanced diet: A diet rich in antioxidant vitamins (C and E), Zn^2+^, and Se can mitigate the harmful effects of heavy metals by reducing oxidative stress and supporting detoxification [207,208]. Optimal selenium levels can reduce toxicity caused by AsIII and Cd^2+^ [209]. Ca^2+^ and Mg^2+^ ions compete with Pb^2+^ and Cd^2+^ for binding at enzymatic sites, reducing their toxicity [210].
- Increasing fiber intake: Dietary fiber can bind heavy metals like Pb^2+^, Cd^2+^, and AsIII in the digestive tract and aid in their excretion [211].
- Optimal hydration: Adequate water intake is essential for supporting the body’s natural detoxification processes, facilitating the excretion of heavy metal ions like AsIII and Hg^2+^.

Globally, policies aimed at reducing heavy metal pollution are important to protect environmental health, ensure sustainable ecosystems, and safeguard human well-being. Such policies focus on limiting the release of toxic metals from industrial, agricultural, and urban activities. These include enforcing stricter regulations on industrial emissions, promoting cleaner production technologies, and improving waste management practices. Reducing heavy metals in agriculture, such as minimizing the use of contaminated fertilizers and pesticides containing Pb^2+^ and Cd^2+^, is also a critical area for policy action [212,213].

Reducing heavy metal pollution sustainably requires a combination of prevention, mitigation, and remediation strategies. Prevention involves minimizing the release of heavy metals through cleaner production processes, adopting green technologies, and transitioning to renewable energy sources. Mitigation includes implementing stricter regulations on industrial discharges, promoting the use of non-toxic alternatives in agriculture, and encouraging the recycling of electronic and hazardous waste. Remediation strategies focus on eco-friendly methods, such as phytoremediation and bioremediation, where plants and microorganisms remove or neutralize heavy metal contaminants like AsIII, Pb^2+^, and Cd^2+^ from contaminated environments. Public awareness campaigns also play a significant role in promoting sustainable practices and reducing pollution at its source [214].

Certain protective factors in foods can help reduce heavy metal toxicity by enhancing detoxification and mitigating cellular damage at the enzymatic level. Antioxidants such as vitamins C and E neutralize ROS generated during heavy metal exposure, thus reducing oxidative stress caused by metals like AsIII, Pb^2+^, and Cd^2+^. At the enzymatic level, antioxidants like glutathione, found in foods such as garlic and cruciferous vegetables, support detoxifying enzymes like GPx and glutathione-S-transferase, which help neutralize and eliminate toxic metals. Sulfur-containing compounds, present in foods like onions and garlic, aid in glutathione synthesis and promote the excretion of heavy metals, such as Hg^2+^, Cd^2+^, and Pb^2+^. Fiber-rich foods, like fruits, vegetables, and whole grains, can bind to heavy metals in the digestive tract, reducing absorption and promoting their elimination through feces. Selenium, from sources like Brazil nuts and fish, plays a synergistic role with antioxidants in neutralizing mercury and other metals, while zinc helps maintain cellular integrity and supports enzymatic functions that repair metal-induced damage [215,216,217].

Sulfur-rich mineral waters, which contain sulfur compounds like hydrogen sulfide, may help reduce the toxic effects of heavy metal poisoning. Sulfur supports the formation of glutathione, a potent antioxidant that neutralizes heavy metals like Hg^2+^, Cd^2+^, and Pb^2+^ by facilitating their excretion. Sulfur compounds in these waters may also enhance liver function, supporting detoxification processes. However, sulfur-rich mineral waters should be viewed as complementary to other treatment methods, such as dietary adjustments and medical interventions, for addressing heavy metal toxicity [218,219].

Maritime areas are classified according to consumer safety standards to ensure the quality and safety of seafood products, which may be contaminated with heavy metals such as Hg^2+^, Cd^2+^, and Pb^2+^. Regulatory frameworks, including the European Union’s Common Fisheries Policy (CFP), help monitor and manage these risks. Consumers should prioritize smaller fish species, which accumulate fewer heavy metals, and choose sustainably sourced seafood certified by reputable organizations, such as the Marine Stewardship Council (MSC) [220,221,222].

Heavy metal poisoning, particularly from Pb^2+^, Hg^2+^, Cd^2+^, and AsIII, remains a significant global health issue. Regions with the highest incidence of heavy metal poisoning-related deaths include South Asia, Sub-Saharan Africa, and parts of Southeast Asia, largely due to industrial pollution, mining, and unsafe water sources. Global efforts to improve regulatory policies, increase environmental monitoring, and reduce exposure through safer industrial practices and public health interventions are essential to mitigate heavy metal-related health risks [223,224,225,226,227,228,229,230].

The detection of heavy metal contamination is a critical aspect of environmental monitoring, yet many reviews fail to adequately explore the advanced analytical techniques available for accurate and sensitive assessment. Among the most widely employed methods, Inductively Coupled Plasma Mass Spectrometry (ICP-MS) stands as the gold standard for detecting trace amounts of toxic metals, including AsIII, AsV, Cd^2+^, MeHg (CH_3_Hg^+^), Hg^2+^, and Pb^2+^ [231,232], due to its unmatched sensitivity and ability to analyze complex environmental and biological samples. In addition to ICP-MS, emerging techniques such as graphene-based sensors offer high sensitivity and the rapid detection of metals, including lead and mercury, thanks to their excellent conductivity and large surface area. X-ray fluorescence (XRF) provides a non-destructive means to analyze metal concentrations in environmental matrices like soil and water, offering the advantage of quick, on-site assessments without sample preparation [233]. Furthermore, atomic absorption spectroscopy (AAS), particularly graphite furnace AAS, remains a widely used technique for metals like Cd^2+^ and As (AsIII and AsV), enabling precise measurement even at low concentrations [234]. In parallel, electrochemical biosensors, incorporating nanomaterials and enzymatic detection, have emerged as promising tools for the real-time, portable monitoring of heavy metal contamination in water and food [235]. These technologies not only enhance the accuracy and efficiency of detection but also expand the scope of environmental monitoring, enabling more comprehensive assessments of heavy metal exposure. Integrating these advanced methods into research and regulatory frameworks is essential for more effective risk assessment and the development of strategies to mitigate public health risks associated with toxic metals.

### 2.3. Establishing Causality Between Human Exposure to Toxic Metals and the Etiology of Neurological and Neurodevelopmental Disorders

One of the foremost scientific challenges in neurotoxicology is determining whether chronic exposure to toxic metals can causally contribute to the etiology of grievous human diseases such as Alzheimer’s disease (AD), Parkinson’s disease (PD), and autism spectrum disorder (ASD). While epidemiological studies have historically focused on correlations, a more robust understanding of causality demands integration across disciplines, longitudinal studies, molecular toxicology, bioinorganic chemistry, and genetic epidemiology.

A growing body of epidemiological studies has documented associations between the exposure to heavy metals and increased incidence of neurodegenerative diseases. For instance, a nationwide geospatial analysis in the United States found statistically significant correlations between the prevalence of AD, PD, and amyotrophic lateral sclerosis (ALS) and elevated levels of Pb^2+^, Cd^2+^, and AsIII in environmental matrices such as topsoil, sewage sludge, and infant blood. Notably, Pb^2+^ in infant blood was found to be strongly associated with the prevalence of neurodevelopmental disorders (NDDs), with an odds ratio of 4.08 (95% CI: 3.14–5.31), although exact concentration ranges were not specified [236].

Experimental evidence supports the plausibility of these associations through established biological mechanisms. A study using in vitro hippocampal HT-22 cell models exposed to Pb^2+^, AsIII, and MeHg found differential expression in key proteins related to mitochondrial electron transport chain (ETC) function, oxidative stress response, proteasomal degradation, and mRNA splicing, pathways that are central to the pathophysiology of both AD and PD [237]. Notably, ETC-related proteins such as CcO subunits and F_1_F_0_-ATPase showed marked dysregulation, indicating compromised ATP production and elevated ROS generation. Oxidative stress markers including SOD (SOD1 and SOD2), glutathione reductase, and monoamine oxidase A (MAO-A) were also altered. In the proteasome pathway, changes in ubiquitin-related enzymes such as UBA1, NEDD4, and UCHL3 were observed, implicating disrupted protein turnover. Moreover, splicing-related proteins including U1-70K and serine/arginine-rich splicing factors (e.g., SFRS1, SFRS2) were differentially expressed, suggesting impaired RNA processing. Although specific metal concentrations used in vitro were not universally reported, effects were observed in the micromolar range (0.24–24 μM).

Further mechanistic support comes from genomic toxicology. Pb^2+^ exposure was shown to induce de novo copy number alterations (CNAs) in zebrafish fibroblast cells, with dose-dependent genomic instability observed at concentrations of 0.24, 2.4, and 24 μM Pb^2+^. A total of 72 CAN amplifications were identified, many of which mapped to genomic regions encompassing genes involved in neurodevelopment and neurodegeneration. Notably, 9 out of 12 genes located within these CNAs, including LFNG, PBX4, SLC13A4, DOC2A, INPP5A, PCSK5, RFK, RTN4IP1, and HDC, were found to be functionally connected to the amyloid precursor protein (APP) molecular network, a central element in AD pathogenesis [238]. These particular genes participate in key biological functions such as synaptic transmission, mitochondrial function, transcriptional regulation, and histamine synthesis. For example, RTN4IP1 is associated with mitochondrial respiratory chain regulation and retinal neuronal survival, while PBX4 and PCSK5 play roles in neurodevelopmental transcriptional regulation and proteolytic processing, respectively. The presence of CNAs in these genes suggests a plausible genomic mechanism through which Pb^2+^ exposure may alter gene dosage, disrupt transcriptional stability, and potentially predispose exposed individuals to neurodegenerative diseases. Although no CNAs were found directly within the APP locus itself, the molecular network proximity of these genes underscores the relevance of Pb-induced genomic alterations in AD-related pathways.

Regarding genetic and epigenetic mechanisms, evidence is emerging that heavy metals may also drive disease through genetic predisposition and epigenetic modulation. For example, exposure to Pb^2+^ and AsIII during early development has been linked to persistent epigenetic changes, including histone modifications and altered non-coding RNA expression, that mirror features seen in AD and PD [239].

Autism spectrum disorders (ASD) have also been linked to prenatal and early-life exposure to heavy metals (Pb^2+^ and Cd^2+^). A review of 25 studies on Pb^2+^ exposure and ASD found that 36% reported positive associations, with higher rates for Cd^2+^ (72%). However, many of these studies lack uniform exposure metrics, and only a minority consider sex-specific susceptibility [240].

In conclusion, elucidating the causal relationship between toxic metal exposure and the pathogenesis of neurodegenerative and neurodevelopmental disorders necessitates a rigorous, multidisciplinary framework. Only through the systematic integration of longitudinal epidemiological evidence, validated biomarkers, mechanistic toxicology, and advanced causal inference methodologies can we transition from observed associations to scientifically robust determinations of causality.

### 2.4. Broader Context and Limitations

Climate change is increasingly recognized as a critical factor influencing the mobilization, transformation, and bioavailability of heavy metals in agricultural environments. Rising temperatures, altered precipitation patterns, and shifts in groundwater dynamics can significantly impact the movement of metal contaminants through soil and water systems. For instance, even modest increases in groundwater levels, which is expected in some regions withing the next two decades, can enhance the transport of toxic metals such as AsIII, AsV, and Pb^2+^ by a factor of 2 to 10, posing elevated risks to drinking water and crop contamination [241]. Climate conditions such as increased rainfall and temperature have also been shown to promote the redistribution of metals into more bioavailable and potentially hazardous forms, particularly in subtropical agricultural watersheds [242].

The intersection of climate change with soil contamination thus underscores the urgent need for integrated monitoring systems and adaptive management strategies to safeguard agricultural productivity and public health [243]. Meanwhile, global trade and transboundary food distribution may inadvertently contribute to the dissemination of contaminated products, raising additional concerns for food safety surveillance.

In parallel, this review acknowledges certain inherent limitations. Many of the cited studies vary in terms of exposure metrics, model organisms, and outcome measures, making direct comparisons difficult. Furthermore, longitudinal human data remain limited, particularly for chronic low-dose exposures and mixtures. Addressing these gaps will require harmonized methodologies, standardized biomonitoring tools, and interdisciplinary collaboration that spans molecular toxicology, epidemiology, and environmental policy.

### 2.5. Future Perspectives

Despite significant advancements in elucidating the toxicological pathways and health consequences of heavy metal exposure, considerable gaps remain that hinder effective public health interventions and regulatory frameworks.

First, there is an urgent need for longitudinal human studies with clearly defined exposure windows and comprehensive dose-response assessments. The studies should incorporate individual susceptibility markers, including genetic polymorphisms and epigenetic profiles, to better understand inter-individual variability in response to heavy metals [244,245]. Existing research is often cross-sectional, limiting causal inference and overlooking temporal dynamics in exposure and response.

Second, future investigations should prioritize the integration of advanced analytical methodologies for metal speciation and biomonitoring. Techniques such as high-resolution inductively coupled plasma mass spectrometry (ICP-MS), HPLC-IPC-MS, and single-cell metal imaging allow for the discrimination between toxic and biologically inert metal species, thereby enhancing risk assessment precision.

Third, a shift towards studying metal mixtures, rather than isolated elements, is critical. Metals often co-occur in the environment and exhibit synergistic or antagonistic interactions that can significantly alter toxicity thresholds, particularly in sensitive populations like children, pregnant individuals, and the elderly.

Furthermore, interdisciplinary approaches that integrate neurotoxicology, exposomics, and systems biology will be vital for the discovery of early biomarkers of exposure and effect. Multi-omics strategies, including transcriptomics, proteomics, and metabolomics, can guide targeted therapeutic and preventive strategies.

Finally, public health and environmental policy must evolve to include dynamic surveillance systems for heavy metal contamination, particularly in vulnerable communities. Real-time biomonitoring, coupled with community education on safe agricultural practices, industrial regulation, and dietary modifications, can significantly mitigate exposure risks. Emphasizing a One Health approach (which integrates human, animal, and environmental health) will be critical for the comprehensive management of heavy metal toxicity [246].

## 3. Conclusions

Heavy metal contamination in food systems remains a persistent toxicological and public health concern due to the bioaccumulative and chemically diverse nature of metal species such as AsIII, AsV, Pb^2+^, Cd^2+^, and MeHg. These harmful metal species infiltrate ecosystems via industrial emissions, agrochemicals, and urban waste, disrupting soil microbiota, contaminating water supplies, and entering the food chain.

Addressing these risks requires an integrated framework that prioritizes accurate speciation analysis, dose-response assessments, and mechanistic toxicology. Also, strengthening regulatory standards, enhancing surveillance through molecular and biosensor technologies, and promoting phytoremediation and non-toxic agricultural inputs are essential to reduce environmental burden and health effects.

In summary, mitigating the complex toxicological impact of heavy metal species on food safety and human health demands coordinated action across environmental policy, molecular toxicology, and risk communication. Only through such interdisciplinary collaboration can we ensure long-term ecological stability and food system resilience.

## Figures and Tables

**Table 1:** Maximum level for various heavy metals across different food types and drinking water.

Heavy Metal	Food Type	Maximum Level (mg/kg or mg/L)	Regulatory Agency	Reference
**Lead (Pb)**	Cereals and grains	0.02 mg/kg	FDA, EFSA	[9,10]
	Vegetables	0.10 mg/kg	EFSA	[10]
	Fruits	0.10 mg/kg	EFSA	[10]
	Meat and offal	0.10 mg/kg	EFSA	[10]
	Fish and seafood	0.30 mg/kg	EFSA	[10]
	Milk and dairy products	0.02 mg/kg	EFSA	[10]
	Bottled water/drinking water	0.005 mg/L (5 ppb);	FDA	[11]
		0.01 mg/L (EPA)	EPA, WHO	[12,13]
	Juice	0.05 mg/L (50 ppb)	FDA	[14]
	Candy	0.1 mg/kg (100 ppb)	FDA	[9]
	Baby foods (fruits, vegetables, yogurts, dry cereals)	0.01 mg/kg (10 ppb)	FDA (proposed)	[15]
**Cadmium (Cd)**	Cereals and grains	0.1 mg/kg	EFSA	[10]
	Leafy vegetables	0.20 mg/kg	EFSA	[10]
	Root and tuber vegetables	0.10 mg/kg	EFSA	[10]
	Legumes	0.10 mg/kg	EFSA	[10]
	Fish and seafood	0.05 mg/kg	EFSA	[10]
	Meat (excluding offal)	0.05 mg/kg	EFSA	[10]
	Bottled water/drinking water	0.005 mg/L (5 ppb);	FDA	[11]
		0.003 mg/L (EPA)	EPA, WHO	[12,13]
**Mercury (Hg)**	Fish and seafood (general)	0.5 mg/kg	Commission Regulation (EU)	[16]
	Large predatory fish (e.g., tuna, shark)	1.0 mg/kg	Commission Regulation (EU)	[16]
	Bottled water/drinking water	0.002 mg/L (2 ppb);	FDA	[11]
		0.001 mg/L (EPA)	EPA, WHO	[12,13]
	Fish (e.g., king mackerel, shark, swordfish, tilefish)	Avoid consumption by pregnant women and young children	FDA	[15]
**Arsenic (As)**	Rice and rice-based products	0.1 mg/kg	EFSA	[10]
	Bottled water/drinking water	0.01 mg/L (10 ppb)	FDA, EPA, WHO	[11,12,13]
	Rice cereals for infants	0.1 mg/kg (100 ppb)	FDA	[15]
**Chromium (Cr VI)**	All food (total Cr)	0.05 mg/kg	EFSA	[10]
**Nickel (Ni)**	Chocolate and cocoa products	0.80 mg/kg	EFSA	[10]
	Nuts and seeds	0.50 mg/kg	EFSA	[10]
	Cereal-based products	0.20 mg/kg	EFSA	[10]

**Table 2:** Mercury levels in food products across different countries.

Heavy Metal	Country	Contaminated Food	Identified Level	Reference
Mercury	Canada	Arctic char	0.92 mg/kg	[98]
Mercury	Canada	Harp seal meat	1.06 mg/kg	[98]
Mercury	Cyprus	UHT Milk	3.66 mg/kg	[99]
Mercury	Italy	Different species of fish	0.7–1 mg/kg	[100]
Mercury	Italy	Pork meat products (baked ham, raw ham, mortadella, cured sausage, salami)	0.01–0.02 mg/kg	[101]
Mercury	Italy	Shellfish	0.023–0.150 mg/kg	[100]
Mercury	Norway	Fish burgers/cakes	0.016–0.035 mg/kg	[102]
Mercury	Norway	Saithe products	0.015–0.018 mg/kg	[102]
Mercury	Poland	Freshwater fish	0.063 mg/kg	[103]
Mercury	Poland	Saltwater fish	0.100 mg/kg	[103]
Mercury	Republic of Korea	Fish	0.04–1.5 mg/kg	[104]
Mercury	Republic of Korea	Fishery products (canned)	0.02–0.13 mg/kg	[104]
Mercury	Turkey	Raw cow milk	0.18 μg/kg	[99]
Mercury	USA	Canned tuna (includes tuna in water or oil)	0.096–0.431 mg/kg	[105]

**Table 3:** Cadmium levels in food products across different countries.

Heavy Metal	Country	Contaminated Food	Identified Level	Reference
Cadmium	Africa	Red meat	74.69–94.66 μg/kg	[125]
Cadmium	Bangladesh	Different species of fish	0.01–1.09 mg/kg	[126,127,128]
Cadmium	Bangladesh	Onion	0.2 mg/kg	[129]
Cadmium	Bangladesh	Tomato	0.056–2.39 mg/kg	[130]
Cadmium	Belgium	Chocolate	0.010–0.090 mg/kg	[131]
Cadmium	Belgium	Fish	0.001–1.5 mg/kg	[131]
Cadmium	Belgium	Potatoes	0.005–0.140 mg/kg	[131]
Cadmium	Bolivia	Rice grain	23.05 μg/kg	[132]
Cadmium	Canada	Beaver kidney	21.6 mg/kg	[98]
Cadmium	Canada	Moose kidney	9.8 mg/kg	[98]
Cadmium	Canada	Mussels	0.56 mg/kg	[98]
Cadmium	Canada	Oysters	1.85 mg/kg	[98]
Cadmium	China	Red seaweed	0.408–1.05 mg/kg	[133]
Cadmium	Cyprus	Halloumi cheese	44.33 μg/kg	[134]
Cadmium	Cyprus	Olive oil	0.02–0.09 mg/kg	[135]
Cadmium	Cyprus	UHT milk	5.00 μg/kg	[134]
Cadmium	Germany	Cereals	29.18 μg/kg	[136]
Cadmium	Germany	Seeds and oleaginous fruits	20.33 μg/kg	[136]
Cadmium	Ghana	Rice grain	13.4 μg/kg	[132]
Cadmium	Malaysia	Chicken liver	0.01–0.22 mg/kg	[137]
Cadmium	Malaysia	Pig liver	0.00–0.34 mg/kg	[137]
Cadmium	India	Rice grain	27. 55 μg/kg	[132]
Cadmium	Italy	Cereals	6.24–54.52 μg/kg	[138]
Cadmium	Italy	Dry foods	0.00–272.50 μg/kg	[138]
Cadmium	Italy	Fish and seafood	0.13–136.71 μg/kg	[138]
Cadmium	Italy	Meat	0.07–25.72 μg/kg	[138]
Cadmium	Japan	Brown seaweed	0.343–1.55 mg/kg	[133]
Cadmium	Japan	Red seaweed	0.089–0.877 mg/kg	[133]
Cadmium	Norway	Fish fingers	0.005–0.007 mg/kg	[102]
Cadmium	Norway	Spread canned mackerel in tomato sauce	0.007–0.016 mg/kg	[102]
Cadmium	Romania	Honey	0.006–0.083 μg/g	[139]
Cadmium	Romania	Wild edible mushrooms	0.33–1.21 μg/g 1.5–6.20 μg/g	[140,141]
Cadmium	Romania	Fish	0.010–0.091 μg/g	[142]
Cadmium	Serbia	Chocolate	0.034 mg/kg	[143]
Cadmium	Serbia	Meat (beef, chicken, pork)	<0.0003 mg/kg	[143]
Cadmium	Serbia	Paprika	0.118 mg/kg	[143]
Cadmium	Serbia	Sugar	0.060 mg/kg	[143]
Cadmium	South Korea	Red seaweed	2.91–3.19 mg/kg	[133]
Cadmium	Spain	Lettuce	<0.005 mg/kg	[144]
Cadmium	Turkey	Raw cow milk	0.53 μg/kg	[145]

**Table 4:** Lead levels in food products across different countries.

Heavy Metal	Country	Contaminated Foodstuff	Identified Level	Reference
Lead	Africa	Red meat	840.64–1094.42 μg/kg	[125]
Lead	Bangladesh	Banana	0.05–0.003 mg/kg	[161]
Lead	Bangladesh	Different species of fish	0.11–12.32 mg/kg	[126,127,128]
Lead	Bangladesh	Tomato	14.15 mg/kg	[130]
Lead	Brazil	Grains, cereals and products	0.056 mg/kg	[162]
Lead	Brazil	Oils and fat spreads	0.078 mg/kg	[162]
Lead	Brazil	Sugar	0.048 mg/kg	[108]
Lead	Canada	Bison meat	0.01 mg/kg	[162]
Lead	Canada	Duck heart	4.67 mg/kg	[98]
Lead	Canada	Squirrel meat	1.46 mg/kg	[98]
Lead	China	Edible fungi	0.240 mg/kg	[163]
Lead	China	Grain, maize	0.02-0.013 mg/kg	[164]
Lead	China	Preserved egg	1.212 mg/kg	[163]
Lead	China	Tea	1.937 mg/kg	[163]
Lead	Cyprus	Olive oil	0.15–1.48 mg/kg	[135]
Lead	England	Coconut oil	0.158 mg/kg	[165]
Lead	England	Olive oil	0.143 mg/kg	[165]
Lead	England	Rapeseed oil	0.181 mg/kg	[165]
Lead	England	Sunflower oil	0.274 mg/kg	[165]
Lead	France	Butter	2.16 μg/kg	[166]
Lead	France	Cheese	6.4 μg/kg	[166]
Lead	France	Croissant-like pastries	8.2 μg/kg	[166]
Lead	France	Sweet and savory biscuits and bars	9.6 μg/kg	[166]
Lead	Italy	Cereals and cereal products	11.427 μg/kg	[167]
Lead	Italy	Citrus fruit	4.618 μg/kg	[167]
Lead	Italy	Dry fruit	32.254 μg/kg	[167]
Lead	Italy	Eggs	0.442 μg/kg	[167]
Lead	Italy	Fish and seafood	18.713 μg/kg	[167]
Lead	Italy	Leafy vegetables	38.953 μg/kg	[167]
Lead	Italy	Meat and meat products	12.520 μg/kg	[167]
Lead	Italy	Milk and dairy products	6.792 μg/kg	[167]
Lead	Italy	Mushrooms	24.997 μg/kg	[167]
Lead	Italy	Nuts and seeds	4.425 μg/kg	[167]
Lead	Italy	Oils and fats	1.658 μg/kg	[167]
Lead	Italy	Pulses	7.615 μg/kg	[167]
Lead	Italy	Red wine	17.164 μg/kg	[167]
Lead	Italy	Sweets, chocolate, cakes	15.306 μg/kg	[167]
Lead	Japan	Brown seaweed	0.106–0.885 mg/kg	[133]
Lead	Japan	Red seaweed	0.123–1.52 mg/kg	[133]
Lead	Malaysia	Cattle liver	0.04–1.52 mg/kg	[137]
Lead	Malaysia	Chicken liver	0.05–0.23 mg/kg	[137]
Lead	Malaysia	Pig liver	0.04–0.13 mg/kg	[137]
Lead	Mexico	Black pepper	0.239 mg/kg	[168]
Lead	Mexico	Infant rice cereal	1.005 mg/kg	[168]
Lead	Mexico	Pre-cooked rice	0.276 mg/kg	[168]
Lead	Mexico	Turmeric	0.176 mg/kg	[168]
Lead	Mexico	Whole wheat bread	0.447 mg/kg	[168]
Lead	Romania	Honey	0.12–0.52 μg/g	[139]
Lead	Romania	Wild edible mushrooms	0.31–0.85 μg/g 0.70–2.80 μg/g	[141] [140]
Lead	Romania	Fish	0.19–0.65 μg/g	[142]
Lead	Serbia	Candy	0.323 mg/kg	[143]
Lead	Serbia	Lettuce	0.080 mg/kg	[143]
Lead	Serbia	Meat (beef, pork, chicken)	0.020–0.098 mg/kg	[143]
Lead	South Korea	Brown seaweed	0.648 mg/kg	[133]
Lead	Spain	Red seaweed	0.123–0.817 mg/kg	[133]

**Table 5:** Arsenic levels in food products across different countries.

Heavy Metal	Country	Contaminated Foodstuff	Identified Level	Reference
Arsenic	Bangladesh	Different species of fish	0.19–0.73 mg/kg	[126,127,128]
Arsenic	Bangladesh	Onion	0.1 mg/kg	[129]
Arsenic	Bangladesh	Potatoes	0.07–1.39 mg/kg	[174]
Arsenic	Bangladesh	Spinach	0.26 mg/kg	[130]
Arsenic	Canada	Crabs	7.83 mg/kg	[98]
Arsenic	Canada	Prawns	8.48 mg/kg	[98]
Arsenic	Canada	Seaweed	31.00 mg/kg	[98]
Arsenic	Chile	Brown seaweed	15.2 mg/kg	[133]
Arsenic	Egypt	White rice	0.01–0.58 mg/kg	[175]
Arsenic	France	White rice	0.09–0.56 mg/kg	[175]
Arsenic	Japan	Brown seaweed	4.1–149 mg/kg	[133]
Arsenic	Japan	White rice	0.07–0.42 mg/kg	[175]
Arsenic	Norway	Fish burgers/cakes	0.37–1.92 mg/kg	[102]
Arsenic	Norway	Fish fingers	1.21–3.01 mg/kg	[102]
Arsenic	Norway	Spread salmon/trout	0.28–0.6 mg/kg	[102]
Arsenic	Portugal	Norway lobster	23.1–51.2 mg/kg	[176]
Arsenic	Portugal	Sardine	4.2–5.6 mg/kg	[176]
Arsenic	Serbia	Canned fish	0.43 mg/kg	[143]
Arsenic	Serbia	Oil and margarine	0.03 mg/kg	[143]
Arsenic	Serbia	Sausage	0.04 mg/kg	[143]
Arsenic	South Korea	Red seaweed	18.4–23.5 mg/kg	[133]
Arsenic	Spain	Red seaweed	34.5 mg/kg	[133]
Arsenic	United Kingdom	Baby rice	0.11 mg/kg	[123]
Arsenic	United States of America	White rice	0.03–0.66 mg/kg	[175]

**Table 6:** Tin levels in food products across different countries.

Heavy Metal	Country	Contaminated Foodstuff	Identified Level	Reference
Tin	France	Preserved foods in unlacquered cans:		[188]
		Tomatoes	46–156 mg/kg	
		Mushrooms	24–45 mg/kg	
		Pineapples	44–136 mg/kg	
		Fruit cocktail	88–107 mg/kg	
		Preserved foods in lacquered cans:		
		Tomatoes	3.2–8.8 mg/kg	
		Mushrooms	0.4–13.4 mg/kg	
		Meats	1.1–9.4 mg/kg	
		Fishes	0.3–0.9 mg/kg	
		Carrots	0.08 mg/kg	
		Alcoholic beverages	<0.003 mg/L	
Tin	Italy	Aged cheese	5.05 μg/kg	[189]
Tin	Italy	Beverages:		[189]
		Coffee and tea	1.81 μg/kg	
		Wines	1.17 μg/kg	
		Spirits and liqueurs	3.58 μg/kg	
		Fruit juices	0.39 μg/kg	
		Soft drinks	0.21 μg/kg	
Tin	Italy	Cereals and cereal products (pasta, rice, bread, salty snacks)	3.6 μg/kg	[189]
Tin	Italy	Meat and meat products (red, white and processed meat)	5.73 μg/kg	[135]
Tin	Italy	Oils and fats	2.04 μg/kg	[189]
Tin	Italy	Potatoes	2.19 μg/kg	[189]
Tin	Italy	Preserved and tinned fish	10.41 μg/kg	[189]
Tin	Italy	Sweets, chocolate, cakes and other pastries	6.01 μg/kg	[189]
Tin	Malaysia	Canned meat and poultry	0.01–0.34 mg/kg	[137]
Tin	Malaysia	Canned seafood	0.00–2.06 mg/kg	[137]
Tin	Malaysia	Canned vegetables	96–937 mg/kg	[137]
Tin	Nigeria	Costa mackerel	5.93–6.01 mg/kg	[190]
Tin	Nigeria	Freshwater fish	0.91–1.04 mg/kg	[190]
Tin	Nigeria	Heineken beer	<0.01 mg/kg	[191]
Tin	Nigeria	Saltwater fish	0.79–1.08 mg/kg	[190]
Tin	Nigeria	Titus sardines	11.94–12.01 mg/kg	[190]
Tin	UK	Corned beef	19 mg/kg	[192]
Tin	UK	Fruits:		[192]
		Orange	124 mg/kg	
		Grapefruit	112 mg/kg	
		Pear	64 mg/kg	
		Red fruits (raspberries, blackcurrants, red plums, blackberries)	37 mg/kg	
Tin	UK	Potatoes	13 mg/kg	[192]

## Data Availability

The original contributions presented in the study are included in the article; further inquiries can be directed to the corresponding author.
